# Consecuencias de los conflictos armados en la salud mental de niños y adolescentes: revisión de revisiones de la literatura

**DOI:** 10.7705/biomedica.5447

**Published:** 2021-09-22

**Authors:** Sandra Piñeros-Ortiz, Jaime Moreno-Chaparro, Nathaly Garzón-Orjuela, Zulma Urrego-Mendoza, Daniel Samacá-Samacá, Javier Eslava-Schmalbach

**Affiliations:** 1 Grupo de Investigación Violencia y Salud, Facultad de Medicina, Universidad Nacional de Colombia, Bogotá, D.C., Colombia Universidad Nacional de Colombia Grupo de Investigación Violencia y Salud Facultad de Medicina Universidad Nacional de Colombia BogotáD.C Colombia; 2 Departamento de Psiquiatría, Facultad de Medicina, Universidad Nacional de Colombia, Bogotá, D.C., Colombia Universidad Nacional de Colombia Departamento de Psiquiatría Facultad de Medicina Universidad Nacional de Colombia BogotáD.C Colombia; 3 Grupo de Investigación en Equidad en Salud, Facultad de Medicina, Universidad Nacional de Colombia, Bogotá, D.C., Colombia Universidad Nacional de Colombia Grupo de Investigación en Equidad en Salud Facultad de Medicina Universidad Nacional de Colombia BogotáD.C Colombia; 4 Escuela de Medicina, Facultad de Medicina, Universidad Nacional de Colombia, Bogotá, D.C., Colombia Universidad Nacional de Colombia Escuela de Medicina Facultad de Medicina Universidad Nacional de Colombia BogotáD.C Colombia; 5 Departamento de Salud Pública, Facultad de Medicina, Universidad Nacional de Colombia, Bogotá, D.C., Colombia Universidad Nacional de Colombia Departamento de Salud Pública Facultad de Medicina Universidad Nacional de Colombia BogotáD.C Colombia; 6 Hospital Universitario Nacional de Colombia, Universidad Nacional de Colombia, Bogotá, D.C., Colombia Universidad Nacional de Colombia Hospital Universitario Nacional de Colombia Universidad Nacional de Colombia BogotáD.C Colombia

**Keywords:** niños, adolescentes, salud mental, conflictos armados, violencia, guerra, Child, adolescent, mental health, armed conflicts, violence, warfare

## Abstract

**Introducción.:**

Los conflictos armados dejan consecuencias evidentes en la salud mental de la población infantil y adolescente. En ese marco, se ha documentado una serie de situaciones que tienen como factor común la vulnerabilidad de esta población y las afectaciones biopsicosociales significativas.

**Objetivo.:**

Determinar y sintetizar las diferentes consecuencias de los conflictos armados en la salud mental de la población infantil y adolescente.

**Materiales y métodos.:**

Se realizó una búsqueda sistemática exhaustiva de revisiones bibliográficas hasta julio de 2019 en las bases de datos MEDLINE (Ovid), EMBASE, Cochrane Central Register of Controlled Trials, LILACS y otras. Se seleccionaron los artículos y se analizaron de forma narrativa sus características, objetivos y consecuencias en salud mental en tres momentos: antes del conflicto, durante el conflicto y en el posconflicto.

**Resultados.:**

De un total de 587 artículos potencialmente relevantes, se seleccionaron 72. En los estudios sobre el periodo anterior al conflicto, se detallaron experiencias psicológicas y síntomas somáticos anticipatorios. Durante el conflicto, se evidenciaron síntomas regresivos, conductuales y cognitivos, como enuresis, miedo, tristeza, agresión, hiperactividad e inatención, entre otros. Además, se establecieron consecuencias directas, como trastornos de adaptación, depresión, ansiedad y, en mayor medida, estrés postraumático. Por último, en el posconflicto, se recopiló la información sobre los procesos de transmisión de las consecuencias y la resiliencia. Por otro lado, se profundizó en las consecuencias potenciales en el desarrollo biopsicosocial, la moralidad, la identidad, el contexto, la cultura, la educación y la sociedad.

**Conclusiones.:**

Las consecuencias de los conflictos armados en la salud mental se inscriben en un proceso complejo que se expresa en función de la etapa evolutiva de la exposición, del tiempo del conflicto armado y de los factores contextuales.

## Abstract (en)

**Introduction:**

Armed conflicts affect the mental health of children and adolescents. Their outcomes in these populations have been documented identifying vulnerability and significant biopsychosocial damage as the most common factors.

**Objective:**

To identify and synthesize the mental health consequences of armed conflicts in children and adolescents.

**Materials and methods:**

We carried out a comprehensive and systematic search of reviews published until July 2019 in the MEDLINE (Ovid), EMBASE, Cochrane Central Register of Controlled Trials, and LILACS databases, as well as in additional sources. The information was retrieved and analyzed narratively by describing the characteristics and objectives of the studies and the mental health consequences of armed conflicts in three periods of time: pre-armed conflict, during the armed conflict, and post-conflict.

**Results.:**

Out of 587 potentially relevant studies, we finally selected 72. In the pre-armed conflict period, we described in detail the psychological experiences and the anticipatory somatic symptoms. During the conflict, we identified regressive, behavioral, and cognitive symptoms such as enuresis, fear, sadness, aggression, hyperactivity, and inattention, among others. Direct mental health consequences such as adjustment disorders, depression, anxiety, and post-traumatic stress were also identified. Finally, in the post-conflict period, we referred to the transmission of mental health consequences and resilience processes. On the other hand, we reviewed in depth the potential consequences of armed conflicts on biopsychosocial development, morality, identity, culture, education, and society.

**Conclusion.:**

The development of mental health consequences due to the exposure to armed conflicts in these populations is a complex process that depends on the stage of the exposure, the length of the conflict, and contextual factors.

Según el reporte *Conflict Barometer* del *Heidelberg Institute for International Conflict Research,* entre el 2018 y el 2019 se registraron 374 conflictos sociopolíticos activos en el mundo, de los cuales más del 57 % se desarrolló de forma violenta ^1^. El Fondo de las Naciones Unidas para la Infancia (UNICEF) señaló, en el 2018, que la violencia extrema contra niños y adolescentes es una constante en los conflictos armados ^2^. La utilización de infantes y adolescentes como escudos humanos y su reclutamiento forzado son tácticas vigentes, a las que se suman otras formas de violencia como el secuestro, el abuso y la explotación sexual, con serias repercusiones para la salud de las víctimas ^2^.

En el marco de los conflictos armados y otras emergencias humanitarias, se emplean de manera complementaria, aunque diferenciada, los conceptos de salud mental y bienestar psicosocial ^3^. Los organismos del sector de la salud suelen preferir la denominación de salud mental, que alude a aquellas condiciones emocionales, cognitivas y conductuales de las personas afectadas que se abordan desde el paradigma biomédico, en tanto que el término bienestar psicosocial se emplea en las ciencias sociales para referirse a aquellas afectaciones que requieren la intervención y la rehabilitación psicosocial para prevenirlas y superarlas ^3^^,^^4^. En suma, cualquier emergencia humanitaria, incluidos los conflictos armados, tiene efectos complejos que mezclan la perspectiva psicosocial y la de salud mental.

Las vivencias inherentes a los conflictos armados, no solo atentan contra la seguridad y la estabilidad de las poblaciones, sino que producen sufrimiento con consecuencias en la salud mental y el bienestar psicosocial ^5^^,^^6^. En la población infantil, estas afectaciones comprometen procesos sociales, productivos, capacidades y habilidades para convivir en la comunidad ^7^. Varios estudios coinciden en que la vulnerabilidad es mayor cuando la exposición ocurre en etapas tempranas de la vida ^6^^,^^8^^-^^11^ como consecuencia de la muerte o la separación forzada de los seres queridos ^12^^-^^15^. Las secuelas físicas de los ataques directos incrementan esta vulnerabilidad ^14^^-^^16^. Algunos factores contextuales que se suman a lo anterior son el aumento de la pobreza y la discriminación ^15^^,^^16^, el acceso deficiente a los servicios públicos, el hacinamiento o el aislamiento ^8^^,^^16^^,^^17^.

Además de la alteración de las competencias de tipo social y productivo en infantes y adolescentes expuestos a conflictos armados, se reportan en los estudios altas tasas de trastornos y síntomas mentales, especialmente del espectro ansioso, depresivo y traumático (trastornos de estrés postraumático). Las dificultades en la atención, la inseguridad, la agresividad, las distorsiones cognitivas y el consumo de sustancias psicoactivas son expresiones de dichos trastornos y de otros problemas psicosociales ^14^^,^^16^. En oposición a estas consecuencias psicopatológicas, algunos estudios plantean potenciales efectos positivos, específicamente la resiliencia ^6^^,^^8^. En las revisiones publicadas, la influencia del contexto social y de otros factores, como la etapa del conflicto sociopolítico y su ubicación geográfica, limitan la interpretación de los resultados ^8^^,^^15^^,^^18^. Para ofrecer un panorama general de tales consecuencias en la salud mental de niños y adolescentes, se llevó a cabo una revisión de revisiones de la literatura, con el fin de sintetizarlas y plantear recomendaciones encaminadas a mejorar la investigación en este campo.

## Materiales y métodos

Se realizó una búsqueda bibliográfica sistemática y exhaustiva, siguiendo las directrices del grupo Cochrane para revisiones sistemáticas ^19^.

### Criterios de inclusión de los estudios

Se consideraron las revisiones narrativas o sistemáticas, con o sin metaanálisis, de estudios cuantitativos o cualitativos que incluyeran a la población infantil o adolescente expuesta a conflictos armados y presentara las consecuencias o afectaciones de su salud mental y desarrollo biopsicosocial. No se restringió por idioma o año de publicación.

### Método de búsqueda de los estudios

La *búsqueda* (anexo 1) incluyó los estudios publicados hasta julio del 2019 en las bases de datos MEDLINE (plataforma Ovid), EMBASE, *Cochrane Database of Systematic Reviews* (plataforma Ovid) y LILACS. Asimismo, se hizo una búsqueda de literatura gris en *Open Grey* y *Google* académico, y se buscó manualmente en los listados de referencias de los estudios incluidos (bola de nieve).

### Tamización y extracción de datos

La selección inicial de estudios a partir de los títulos y res*úmenes* estuvo a cargo de dos revisores de manera independiente con base en los criterios de inclusión. Los desacuerdos se solucionaron por consenso entre los dos, revisando de nuevo los criterios y los estudios. Posteriormente, se hizo una segunda tamización para verificar los criterios de inclusión mediante la revisión del texto completo.

### Síntesis de la evidencia

Debido a la gran heterogeneidad de los estudios, no fue posible combinar los resultados de las revisiones sistemáticas. Por lo tanto, se elaboró una síntesis narrativa de los hallazgos centrada en la recolección de la mayor cantidad de información sobre el tema, con el fin de determinar el conjunto de saberes que se ha ido construyendo a lo largo del tiempo, el cual se refleja en la recopilación y síntesis juiciosa de los estudios secundarios sobre el tema. Por ello, y teniendo en cuenta el enfoque temático, se decidió descartar la evaluación de la calidad de la evidencia privilegiando el conocimiento.

La síntesis se consolidó a partir del concepto ampliado de salud mental, que abarca el paradigma biomédico y el de bienestar psicosocial, exponiendo en cada uno las afectaciones evidenciadas en los estudios consultados. Los hallazgos se organizaron en torno a los momentos detectables de los conflictos armados en los que las consecuencias aparecían como significativas. En un primer momento, correspondiente al periodo previo al conflicto, estas consecuencias se relacionan con la representación anticipatoria de los eventos traumáticos asociados. Con el inicio del conflicto, se desarrolla un nuevo momento en el cual se consolidan diferentes consecuencias que, posteriormente, evolucionan en un tercer momento, el del posconflicto.

## Resultados

**Figura 1:**
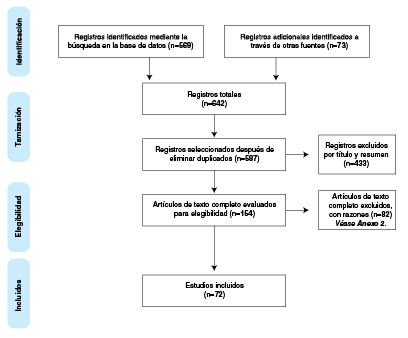
Diagrama de flujo *(Preferred Reporting Items for Systematic Reviews and Meta-Analyses,* PRISMA©)

Se encontraron 587 estudios potencialmente relevantes después de eliminar los duplicados. Después de la revisión del texto completo mediante la metodología PRISMA ^20^, se excluyeron 82 referencias (figura 1, anexo 2); finalmente, se incluyeron 72 publicaciones para la síntesis de evidencia. Las características generales de los estudios incluidos se presentan en el cuadro 1.

**Cuadro 1:** Características generales de los estudios incluido**s**

	Autor y referencia	Tipo de revisión	Tipos de estudios	Número de estudios incluidos	Población
1	Gibson K (1989)	Narrativa	Todos los referentes a la temática	No reportado, tomaron 70 referencias en total.	Víctimas de conflicto
2	Jensen PS, Shaw J (1993)	Narrativa	Todos los estudios y literatura sobre la existencia, frecuencia y tipo de problemas sociales, emocionales y de comportamiento	No reportado, tomaron 80 referencias en total.	Víctimas de conflicto
3	Elbedour S, Ten Bensel R, Bastien DT (1993)	Narrativa	Estudios que revisan la interacción de factores sociales, biológicos e individuales en la intensidad del trauma	No reportado, tomaron 65 referencias en total.	Víctimas de conflicto
4	Fox PG, Cowell JM, Montgomery AC (1994)	Sistemática	Diseños cuasiexperimentales o experimentales. Todos los estudios fueron transversales y retrospectivos y se publicaron entre 1983 y 1992.	12 incluidos	Refugiados del sudeste asiático
5	Rousseau C (1995)	Narrativa	Todos los referentes a la temática	No reportado, tomaron 108 referencias en total.	Refugiados
6	Summerfield D (2000)	Narrativa	Todos los referentes a la temática	No reportado, tomaron 28 referencias en total.	Víctimas de conflicto
7	Davis L, Siegel LJ (2000)	Narrativa	Todos los referentes a la temática, específicamente a TEPT	No reportado, tomaron 117 referencias en total.	Víctimas de conflicto
8	Berman H (2001)	Narrativa	Todos los referentes a la temática, específicamente a TEPT	No reportado, tomaron 73 referencias en total.	Refugiados
9	Mandalakas AM (2001)	Narrativa	Estudios de los efectos a largo plazo	No reportado, tomaron 40 referencias en total.	Expuestos a emergencias humanitarias complejas
10	Fazel M, Stein A (2002)	Narrativa	Todos los referentes a la temática	No reportado, tomaron 55 referencias en total.	Refugiados
11	Hoven CW, Duarte CS, Mandell DJ (2003)	Narrativa	Todos los referentes a la temática	No reportado, tomaron 55 referencias en total.	Refugiados
12	van Ijzendoorn MH, Bakermans-Kranenburg MJ, Sagi-Schwartz A (2003)	Sistemática	Todos los referentes a la temática	32 incluidos	Víctimas de conflicto
13	Pfefferbaum B, Pfefferbaum RL, Gurwitch RH, Nagumalli S, Brandt EN, Robertson MJ, *et al*. (2003)	Narrativa	Todos los referentes a la temática con énfasis en salud mental, servicios para niños y ambiente posterior al ataque	No reportado, tomaron 43 referencias en total.	Víctimas de conflicto
14	DeRanieri JT, Clements PT, Clark K, Kuhn DW, Manno MS (2004)	Narrativa	Todos los referentes a la temática	No reporta, tomaron 34 referencias en total.	Víctimas de conflicto
15	Barenbaum J, Ruchkin V, Schwab-Stone M (2004)	Narrativa	Todos los referentes a la temática	No reportado, tomaron 207 referencias en total.	Víctimas de conflicto
16	Lustig SL, Kia-Keating M, Knight WG, Geltman P, Ellis H, Kinzie JD, *et al*. (2004)	Narrativa	Todos los referentes a la temática	No reportado, tomaron 105 referencias en total.	Refugiados
17	Pine DS, Costello J, Masten A (2005)	Narrativa	Todos los referentes a la temática	No reportado, tomaron 109 referencias en total.	Víctimas de conflicto
18	Ehntholt KA, Yule W (2006)	Narrativa	Estudios publicados sobre las dificultades de salud mental de los niños y adolescentes refugiados	No reportado, tomaron 111 referencias en total.	Refugiados
19	Moss WJ, Ramakrishnan M, Storms D, Siegle AH, Weiss WM, Lejnev I, *et al*. (2006)	Narrativa	Todos los referentes a la temática	No reportado, tomaron 56 referencias en total.	Víctimas de conflicto
20	Williams R (2006)	Narrativa	Todos los referentes a la temática	No reportado, tomaron 85 referencias en total.	Víctimas de conflicto
21	Williams R (2007)	Narrativa	Todos los referentes a la temática	No reportado, tomaron 94 referencias en total.	Víctimas de conflicto
22	Morris J, van Ommeren M, Belfer M, Saxena S, Saraceno B (2007)	Sistemática	Diseños enfocados a la temática con énfasis en estudios de casos e investigación etnográfica	No reportado, tomaron 89 referencias en total.	Víctimas de conflicto y participación como combatientes
23	Betancourt TS, Khan KT (2008)	Narrativa	Todos los referentes a la temática	No reportado, tomaron 99 referencias en total.	Víctimas de conflicto
24	Neuner F, Catani C, Ruf M, Schauer E, Schauer M, Elbert T (2008)	Narrativa	Estudios longitudinales y de seguimiento	No reportado, tomaron 93 referencias en total.	Víctimas de conflicto
25	Lustig SL, Tennakoon L (2008)	Narrativa	Estudios de narrativas y testimonios con énfasis en experiencia, testimonio y análisis del impacto	14 incluidos	Refugiados
26	Dekel R, Goldblatt H (2008)	Narrativa	Todos los referentes a la temática, específicamente de tipo empírico, clínicos y descriptivos de orden cualitativo y cuantitativo	16 estudios empíricos que describen 17 proyectos	Víctimas de conflicto
27	Qouta S, Punamäki R-L, El Sarraj E (2008)	Narrativa	Todos los referentes a la temática	11 artículos principales y 82 referencias de soporte	Víctimas de conflicto
28	Prasad AN, Prasad PL (2009)	Narrativa	Todos los referentes a la temática	No reportado, tomaron 23 referencias en total.	Víctimas de conflicto
29	Jordans MJD, Tol WA, Komproe IH, De Jong JVTM (2009)	Sistemática	Todos los estudios relevantes al tema incluidos ensayos clínicos aleatorizados, de diseño cuasiexperimental y estudios de caso	66 incluidos	Víctimas de conflicto
30	Persson TJ, Rousseau C (2009)	Sistemática	Todos los referentes a la temática con especificidad en empíricos	7 incluidos	Víctimas de conflicto
31	Sullivan PM (2009)	Sistemática	Todos los referentes a la temática	50 incluidos	Víctimas de conflicto con discapacidades
32	Attanayake V, McKay R, Joffres M, Singh S, Burkle F, Mills E (2009)	Sistemática	Todos los referentes a la temática con énfasis en trastorno de estrés postraumático (TEPT)	17 incluidos	Víctimas de conflicto
33	Torrado M, Camargo M, Pineda N, Bejarano D (2009)	Narrativa -estado del arte	Todos los diseños enfocados a la temática	No reportado, tomaron 33 referencias en total.	Víctimas de conflicto, principalmente < 6 años
34	Steel Z, Chey T, Silove D, Marnane C, Bryant RA, van Ommeren M (2009)	Sistemática	Todos los diseños enfocados a la temática	181 incluidos	Refugiados
35	Crowley C (2009)	Narrativa	Todos los estudios referentes a la temática	No reportado, tomaron 52 referencias en total.	Refugiados
36	Peltonen K, Punamäki RL (2010)	Narrativa	Todos los estudios relevantes en el tema incluidos ensayos clínicos aleatorizados, diseños cuasiexperimentales y estudios de caso	16 estudios, 4 ensayos clínicos aleatorizados, 11 diseños de antes y después, 1 estudio de caso	Refugiados
37	Hernández-Barrera A, Restrepo-Espinosa M (2011)	Narrativa	Todos los diseños referentes a la temática	No reportado, tomaron 51 referencias en total.	Refugiados/desplazamiento
38	White CJ, De Burgh HT, Fear NT, Iversen AC (2011)	Sistemática	Todos los diseños referentes a la temática	9 incluidos	Hijos de militares/víctimas de conflicto
39	Bronstein I, Montgomery P (2011)	Sistemática	Todos los diseños referentes a la temática con énfasis en refugiados	22 estudios	Refugiados
40	Henley J, Robinson J (2011)	Narrativa	Todos los diseños referentes a la temática	No reportado, tomaron 104 referencias en total.	Refugiados
41	Yahav R (2011)	Narrativa	Todos los diseños referentes a la temática	No reportado, tomaron 80 referencias en total.	Víctimas de conflicto
42	Montgomery E (2011)	Narrativa de estudios empíricos	Todos los diseños referentes a la temática con énfasis en estudios cualitativos de familias y, en especial, niños sobrevivientes de tortura	11 estudios específicos	Víctimas de conflicto/ refugiados
43	Bohleber W (2012)	Narrativa	Todos los diseños enfocados a la temáti.ca	No reportado, tomaron 18 referencias en total	Víctimas de conflicto
44	Werner, E. E. (2012)	Sistemática	Todos los diseños enfocados a la temática con énfasis en los de corte transversal	No se menciona el número de estudios incluidos.	Víctimas de conflicto énfasis en II Guerra Mundial
45	Masten AS, Narayan AJ (2012)	Narrativa	Todos los diseños enfocados a la temática	No se menciona el número de estudios incluidos.	Víctimas de conflicto
46	Drury J, Williams R. (2012)	Narrativa	Todos los diseños enfocados a la temática entre 2009 y 2012	No reportado, tomaron 46 referencias en total.	Víctimas de conflicto
47	Dimitry L (2012)	sistemática	Todos los diseños enfocados a la temática con énfasis en edades de 0 a 19 años	No reportado, tomaron 71 referencias en total.	Víctimas de conflicto
48	Reed R V, Fazel M, Jones L, Panter-Brick C, Stein A (2012)	Sistemática	Todos los diseños referentes a la temática	27 incluidos	Víctimas de conflicto
49	Fazel M, Reed R V, Panter-Brick C, Stein A (2012)	Sistemática	Todos los diseños referentes a la temática entre 1980 y 2010	44 incluidos	Víctimas de conflicto/desplazados
50	Betancourt TS, Meyers-Ohki SE, Charrow AP, Tol WA (2013)	Sistemática	Todos los diseños referentes a la temática	40 incluidos	Víctimas de conflicto
51	Forman-Hoffman VL, Zolotor AJ, McKeeman JL, Blanco R, Knauer SR, Lloyd SW, *et al*. (2013)	Sistemática	Diseños referentes a la temática con énfasis en ensayos clínicos aleatorizados controlados y estudios de cohorte	22 incluidos	Víctimas de conflicto entre 0 y 17 años
52	Betancourt TS, McBain R, Newnham EA, Brennan RT (2013)	Sistemática	Diseños referentes a la temática con énfasis en estudios de antes y después, estudios longitudinales, estudios observacionales, ensayos clínicos aleatorizados	22 incluidos	Víctimas de conflicto asociados con las fuerzas y grupos armados
53	Rousseau C, Jamil U, Bhui K, Boudjarane M (2013)	Sistemática con métodos mixtos	Diseños referentes a la temática con énfasis en estudios cuantitativos y cualitativos	21 revisiones, 73 estudios cuantitativos, 13 estudios cualitativos, 122 estudios de intervención	Víctimas de conflicto entre los 0 y 20 años
54	Tol WA, Song S, Jordans MJD (2013)	Sistemática	Diseños referentes a la temática con énfasis en estudios de métodos cualitativos, cuantitativos y mixtos	53 incluidos	Víctimas de conflicto
55	Shaar KH (2013)	Sistemática	Diseños referentes a la temática con énfasis en estudios transversales, validación de escalas y una intervención	11 incluidos	Víctimas de conflicto
56	Pacione L, Measham T, Rousseau C (2013)	Narrativa	Todos los diseños referentes a la temática	No referenciado, tomaron 100 referencias en total.	Refugiados
57	Agazio J, Goodman P, Padden DL (2014)	Narrativa	Diseños referentes a la temática con énfasis en estudios investigativos, teóricos, programáticos y documentos gubernamentales	No reportado, tomaron 100 referencias en total.	Víctimas de conflicto
58	Fu C, Underwood C (2015)	Sistemática	Todos los diseños referentes a la temática	11 incluidos	Víctimas de conflicto
59	Foster H, Brooks-Gunn J (2015)	Narrativa	Todos los diseños enfocados a la temática	20 incluidos	Víctimas de conflicto
60	Rosshandler Y, Hall BJ, Canetti D (2016)	Sistemática	Todos los diseños referentes a la temática	20 incluidos	Víctimas de conflicto con énfasis en adolescentes
61	Miller KE, Jordans MJD (2016)	Narrativa	Todos los diseños enfocados a la temática	46 referencias	Víctimas de conflicto
62	Slone M, Mann S (2016)	Sistemática	Todos los diseños referentes a la temática. Se excluyeron capítulos de libro al evidenciar que la información se solapaba con la registrada en artículos publicados	35 incluidos (34 cualitativos y 1 cuantitativo)	Víctimas de conflicto
63	Jordans MJD, Pigott H, Tol WA (2016)	Sistemática	Diseños referentes a la temática con énfasis en estudios de ensayos controlados aleatorios individuales (ECA), controlados aleatorios grupales, controlados, no controlados y de caso	24 incluidos	Víctimas de conflicto
64	Sleijpen M, Boeije HR, Kleber RJ, Mooren T (2016)	Sistemática	Diseños referentes a la temática con énfasis en estudios cualitativos o de métodos mixtos	26 incluidos	Víctimas de conflicto énfasis en edad (10-20 años)
65	Hassan G, Ventevogel P, Jefee-Bahloul H, Barkil-Oteo A, Kirmayer LJ (2016)	Narrativa	Todos los diseños enfocados a la temática	No reportado, tomaron 111 referencias en total.	Refugiados/desplazados
66	Neal S, Stone N, Ingham R (2016)	Sistemática	Todos los diseños enfocados a la temática	19 incluidos	Víctimas de conflicto, énfasis en mujeres
67	Saltzman LY, Solomyak L, Pat-Horenczyk R (2017)	Narrativa	Todos los diseños enfocados a la temática	No reportado, tomaron 89 referencias en total.	Víctimas de conflicto
68	Stough LM, Ducy EM, Kang D (2017)	Narrativa	Todos los diseños enfocados a la temática	92 referencias	Víctimas de conflicto con discapacidad
69	Anagnostopoulos DC, Giannakopoulos G, Christodoulou NG (2017)	Narrativa	Todos los diseños enfocados a la temática	No reportado, tomaron 45 referencias en total.	Víctimas de conflicto
70	Brown FL, de Graaff AM, Annan J, Betancourt TS (2017)	Sistemática	Diseños referentes a la temática con énfasis en estudios de ensayos controlados aleatorios y ensayos controlados	28 incluidos	Víctimas de conflicto
71	Kadir A, Shenoda S, Goldhagen J (2018)	Narrativa	Todos los diseños enfocados a la temática	No reportado, tomaron 172 referencias en total.	Víctimas de conflicto
72	Fajardo-Mayo MA, Ramírez-Lozano MP, Suescún V, Isabel M, Ospina-Alvarado MC (2018)	Sistemática	Todos los diseños enfocados a la temática con énfasis en primera infancia	15 incluidos, 35 descartados	Víctimas de conflicto

TEPT: trastorno de estrés postraumático

**Figura 2:**
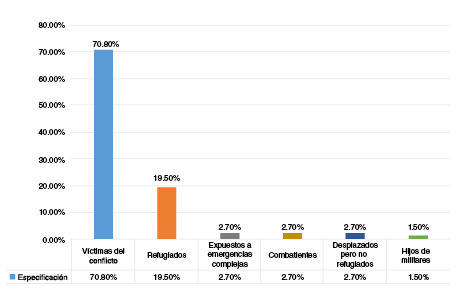
Especificidades de la población

El periodo de publicación de los estudios abarcó de 1989 ^21^ a 2018 ^22^^,^^23^. El 61,1 % de ellos correspondió a revisiones de tipo narrativo y el 38,9 %, a revisiones sistemáticas. Los tipos de estudios incluyeron: a) todos los tipos de estudios cuantitativos y cualitativos; b) estudios exclusivamente cuasiexperimentales o experimentales, transversales o retrospectivos; c) estudios específicos de seguimiento a largo plazo, y d) estudios etnográficos y de casos ^24^. Las especificidades de las poblaciones abordadas en los estudios se detallan en la figura 2; además, en el cuadro 2 se puntualizan los estudios incluidos.

**Cuadro 2:** Descripciones y detalles de los estudios.

	Autor y referencia	Objetivo del estudio	Desenlace o consecuencia en salud mental	Conclusiones
1	Gibson K (1989)	Evaluar la utilidad de la literatura sobre niños en situaciones de guerra y desastres	Existe una distinción entre reacciones crónicas y agudas; las crónicas caracterizadas por síntomas debilitantes y persistentes, las agudas por reacciones normales frente a la exposición a eventos altamente estresantes	El contexto social y familiar es importante en el afrontamiento de situaciones derivadas del conflicto, se requiere que desde todos los sectores se brinden espacios seguros de diálogo y solución de problemas.
2	Elbedour S, ten Bensel R, Bastien DT (1993)	Revisión de la investigación sobre los niños de la guerra	Algunas de las expresiones que se manifiestan después de la experiencia de guerra son el miedo, la ansiedad, el terror o nerviosismo, la depresión, tristeza, ira, vergüenza y culpa. Entre los problemas comportamentales se encuentran agresión, violencia y hostilidad.	Los niños de la guerra pasan por una serie de reacciones emocionales a la situación y sus cambios emocionales no pueden entenderse por fuera de la dinámica familiar y social, y comprenderlo es necesario para el adecuado curso de la terapia.
3	Jensen PS, Shaw J (1993)	Evaluar los efectos de los factores estresantes relacionados con la guerra en niños y adolescentes	Los niños y niñas de la guerra manifiestan comportamientos regresivos, agresión episódica, trastornos psicofisiológicos, cambios en el rendimiento escolar, cambios de personalidad y diversos tipos de depresión y ansiedad.	Las intervenciones deben guiarse de manera individual (ventilación, canalización de la agresión, exposición gradual in vivo a situaciones temidas y reevaluación cognitiva, comunitaria y familiar).
4	Fox PG, Cowell JM, Montgomery AC (1994)	Aclarar la investigación centrada en las necesidades de un grupo de refugiados del sudeste asiático	El estrés y la depresión de los padres puede tener un impacto negativo en el ajuste psicosocial de los niños y en su logro académico.	La discriminación y un ambiente inadecuado en la posguerra pueden intensificar eventos traumáticos, por el contrario, un ambiente de apoyo puede mitigarlo.
5	Rousseau C (1995)	Resumir el conocimiento de las manifestaciones del trastorno emocional entre los niños refugiados	La sintomatología relacionada con el conflicto incluye ansiedad, pesadillas, insomnio, enuresis, introversión, depresión, problemas de comportamiento, dificultades académicas, anorexia y problemas somáticos.	Los niños refugiados están en alto riesgo de desarrollar problemas de salud mental debido a los estresores extremos que experimentan en los períodos previos y posteriores a la migración.
6	Davis L, Siegel LJ (2000)	Revisar la presencia de TEPT en relación con niños y adolescentes en situaciones que incluyen el conflicto armado	Los principales síntomas de TEPT fueron pesadillas, respuesta de sobresalto exagerada, conducta de evitación, dificultad para dormir y concentrarse, así como síntomas depresivos como apetito y cambios de peso.	Después del trauma, los niños volvieron a experimentar el evento a través del juego repetitivo, a menudo participando en juegos de secuestro en un esfuerzo por reducir su ansiedad.
7	Summerfield D (2000)	Proporcionar un telón de fondo para encuentros potenciales o reales entre un niño refugiado y profesionales de la salud mental en países occidentales	Es necesario reconocer el peligro de reducir las experiencias de los niños a una cuestión de salud mental y ver los problemas de vulnerabilidad únicamente en términos psicológicos más que sociales.	Al analizar o intervenir es importante tener una visión tan sofisticada como sea posible de las realidades complejas y cambiantes en las que el niño está enmarcado actualmente.
8	Berman H (2001)	Examinar críticamente el énfasis generalizado en la literatura sobre el trastorno de estrés postraumático (TEPT)	Condiciones deplorables de vida, separación de cuidadores, hermanos y padres es más estresante que los bombardeos. A pesar de la protección de sus padres, se presenta una serie de dificultades emocionales y de comportamiento.	Los niños refugiados se ven afectados de múltiples maneras por el conflicto. Para algunos niños y niñas la violencia representa una amenaza directa para su salud y bienestar.
9	Mandalakas AM (2001)	Evaluar la vulnerabilidad del desarrollo a largo plazo encontrados en niños expuestos a emergencias humanitarias complejas	La vulnerabilidad de los niños y niñas puede ser atribuida a un gran número de factores, incluyendo las reservas limitadas de alimentos, lo que incrementa el riesgo de disminuir las funciones cognitivas, comportamentales y psicológicas.	Los síntomas de trauma psicológico en niños y niñas suelen tener una naturaleza diferente a los de los adultos, por lo que se hace preciso prestar atención profesional con recomendaciones ajustadas a la cultura.
10	Fazel M, Stein A (2002)	Revisar la evidencia sobre el impacto del desplazamiento en la salud mental de los niños	Se reportan niveles elevados de morbilidad psicológica entre los niños refugiados, especialmente el trastorno de estrés postraumático, depresión y trastornos de ansiedad. Los estudios de niños refugiados recién llegados muestran tasas de ansiedad del 49 al 69 %.	Los estudios establecieron factores protectores como: (1) un entorno familiar de apoyo; (2) una agencia social externa y (3) una disposición de personalidad positiva. La prevención primaria puede llevarse a cabo en el contexto escolar.
11	Hoven CW, Duarte CS, Mandell DJ (2003)	Resumir los resultados de estudios sistemáticos sobre las reacciones de estrés postraumático en niños después de desastres masivos	Se reportan niveles elevados de síntomas de estrés postraumático con prevalencias de hasta 87 %. La literatura muestra que las reacciones de estrés postraumático no son temporales, sino que persisten en el tiempo.	Se observó de manera consistente en todos los estudios el mayor riesgo de reacciones de estrés postraumático después de un desastre masivo para niñas y niños más pequeños.
12	van Ijzendoorn MH, Bakermans-Kranenburg MJ, Sagi-Schwartz A (2003)	Probar la hipótesis de “traumatización secundaria” en familias sobrevivientes del Holocausto	Estudios clínicos han reportado alteraciones en las relaciones familiares y en el comportamiento por parte de los sobrevivientes del holocausto y de su descendencia.	Posiblemente la "traumatización secundaria" es transmitida de manera no verbal a los niños por madres que sufrieron pérdidas no resueltas de familiares cercanos.
13	Pfefferbaum B, Pfefferbaum RL, Gurwitch RH, Nagumalli S, Brandt EN, Robertson MJ, et al. (2003)	Resumir los estudios que abordan el impacto de los incidentes terroristas en los niños y niñas	Después del ataque del 11/09/2001 se evidenció que el 10,5 % de los niños desarrollaron desórdenes de estrés postraumático y al menos la cuarta parte tenía alteraciones en salud mental. A nivel general los niños presentaron agorafobia (15 %), ansiedad (12 %), desordenes de conducta (11 %), ansiedad generalizada 10 %) y pánico (9 %).	La relación entre la exposición y los síntomas de estrés postraumático deben ser interpretados con cautela; la exposición, la muestra y otras variables podrían alterar los resultados.
14	DeRanieri JT, Clements PT, Clark K, Kuhn DW, Manno MS (2004)	Explorar el impacto potencial de la guerra y el terrorismo en niños e identificar métodos para la educación familiar	Al proporcionar un entorno terapéutico, se debe recordar que los niños atravesarán dos etapas de afrontamiento de eventos traumáticos: aceptación y la formulación de más preguntas, las cuales deben responder de manera asertiva.	Se debe hacer todo lo posible para explicar a los niños lo que ha ocurrido de la manera más directa. Los niños y niñas necesitan garantías sobre su seguridad y la seguridad de los demás durante toda su infancia, incluso en tiempos difíciles.
15	Barenbaum J, Ruchkin V, Schwab-Stone M. (2004)	Revisar la literatura existente sobre enfoques para ayudar a los niños afectados por la guerra	En casos como los niños israelíes el TEPT se presentó en el 22 %, en la guerra libanesa en el 27 %, en el conflicto de Camboya en el 48 %, en niños refugiados de América Central en el 52 %, en Kuwait en el 70 % después de 5 meses de ocupación militar y hasta el 93,8 % en niños desplazados en la guerra de Bosnia.	La guerra produce conflictos mentales en los niños, ya que puede forzarlos a tomar posiciones o actitudes que van en contra de sus principios. Estos pueden ir desde lo que es bueno o malo, el no aceptar la escuela y aceptar la guerra, los héroes y el contexto.
16	Lustig SL, Kia-Keating M, Knight WG, Geltman P, Ellis H, Kinzie JD, et al. (2004)	Revisar las experiencias estresantes y las reacciones al estrés entre los niños y adolescentes refugiados	Los niños nacidos en la fase de vuelo tienden a tener alteraciones importantes en el desarrollo psicológico. En la fase de reasentamiento, los sentimientos de pérdida de su hogar, familiares, amigos y posesiones materiales, hacen que se cree un nuevo lenguaje y cultura que impacta con las culturas y lógicas de otros sitios.	El impacto de la guerra en niños refugiados se manifiesta empíricamente en modelos de enfermedad e incluyen múltiples traumas, retos de aculturación, resiliencia y las formas de vida con la sintomatología.
17	Pine DS, Costello J, Masten A (2005)	Resumir la literatura relevante sobre los efectos del terrorismo en la salud mental de los niños	Un predictor clave y potencialmente modificable de los resultados de los niños parece ser cómo se comportan los adultos; se ha encontrado que las ansiedades de los padres median los efectos del trauma distante en los temores de los niños.	En el trauma masivo, todos los aspectos de la vida de un niño o niña pueden haber colapsado.
18	Ehntholt KA, Yule W (2006)	Identificar y revisar los estudios publicados sobre las dificultades de salud mental de los niños y adolescentes refugiados	Aunque los refugiados jóvenes a menudo son resilientes, muchos experimentan dificultades de salud mental, como TEPT, depresión, ansiedad y dolor, además de aspectos como quejas somáticas, problemas de sueño, trastorno de conducta, retraimiento social, problemas de atención, miedo generalizado, dependencia exagerada, inquietud e irritabilidad.	Las personas que atienden a niños, niñas y adolescentes deben ser conscientes de que, aunque el TEPT se diagnostica con frecuencia, otras dificultades de salud mental, como la depresión, la ansiedad, el dolor, problemas de sueño y escolares, suelen coexistir y no deben pasarse por alto.
19	Moss WJ, Ramakrishnan M, Storms D, Siegle AH, Weiss WM, Lejnev I, et al. (2006)	Identificar las necesidades de investigación y mejorar las pautas para el cuidado de los niños	Los niños expuestos a conflictos armados o duras condiciones de vida de los campos de refugiados tienen altas tasas de problemas psiquiátricos graves. Aunque la mayoría de los estudios informan altas tasas de TEPT, la depresión y la ansiedad pueden afectar a un mayor número de niños y contribuir más a la carga psicológica a largo plazo.	Las necesidades de salud de los niños refugiados no son las mismas que las de los niños desplazados internos, y pueden diferir entre entornos agudos, crónicos y posteriores a emergencias.
20	Williams R (2006)	Identificar los temas de la investigación sobre políticas, prácticas y gobierno corporativo y clínico después de los conflictos	Los impactos directos pueden resultar en el desarrollo de una gama de problemas psicológicos o psiquiátricos Los diagnósticos incluyen: respuestas de estrés agudo y estrés crónico; trastornos psiquiátricos como conducta, ansiedad y trastornos fóbicos, depresión, abuso de sustancias y TEPT.	Los civiles comprenden el 80-90 % de todos los que mueren o son heridos en conflictos, en su mayoría niños y sus madres. Se necesitan enfoques de diagnóstico culturalmente sensibles para evaluar los síntomas del trauma y el deterioro asociado.
21	Williams R (2007)	Resumir la literatura sobre los impactos psicosociales en menores de edad	Un resumen de efectos psicológicos en niños enmarcados en la guerra incluye soledad, miseria, llanto y tristeza; ansiedad; depresión; dificultades para concentrarse, problemas de aprendizaje, entre otros.	El trauma psicológico del evento y sus secuelas pueden dar un golpe a la sensación de seguridad y personalidad, incluidas las fantasías organizativas centrales y las estructuras de significado.
22	Morris J, van Ommeren M, Belfer M, Saxena S, Saraceno B (2007)	Revisar evidencia para la intervención en aspectos mentales y sociales de la salud	Los niños expuestos a eventos catastróficos tienen un mayor riesgo de síntomas de depresión y ansiedad, y TEPT, problemas de comportamiento, disminución del funcionamiento cognitivo y otros signos no clínicos de angustia mental, física y social.	La escuela y otras actividades pueden ser intervenciones en sí mismas, sin necesidad de programas especializados, pues ayudan a reducir la humillación y el estigma, enriqueciendo socialmente las redes de apoyo y proporcionando un espacio seguro.
23	Betancourt TS, Khan KT (2008)	Evaluar la interacción entre el riesgo y los procesos de protección en la salud mental de los niños afectados por la guerra	Algunos mecanismos usados por los "niños resistentes" fueron: el sentido de agencia; inteligencia social, empatía y regulación del afecto; experiencia compartida, características de cuidado y comunidad y conexión; un sentido de futuro, esperanza y crecimiento; una conexión con la espiritualidad.	Se debe prestar especial atención a la capacidad de afrontamiento y la creación de significado en el nivel individual, el papel del apego, la salud del cuidador, los recursos y la conexión en la familia, y el apoyo social disponible en redes sociales pares y extendidas.
24	Neuner F, Catani C, Ruf M, Schauer E, Schauer M, Elbert T (2008)	Describir los fundamentos y el uso de la terapia de exposición narrativa en niños: KidNET (terapia de exposición narrativa)	Reducción clínicamente significativa en los síntomas de TEPT y una mayor efectividad que el asesoramiento de apoyo y la psicoeducación solamente.	KidNET reduce significativamente los síntomas de TEPT incluso en niños gravemente traumatizados por la guerra. También tiene efecto significativo sobre los síntomas de depresión y sobre la ideación suicida.
25	Lustig SL, Tennakoon L (2008)	Resumir la literatura sobre terapias de creación de historias, como la terapia narrativa y las terapias testimoniales	Los niños pequeños, al trabajar en las luchas típicas del desarrollo infantil, usan las historias para trabajar y reelaborar los conflictos psíquicos de manera regular, a menudo pidiendo una y otra vez la misma historia para dormir.	La psicoterapia testimonial, terapia de exposición narrativa, libros de cuentos y dibujos, son posibles en el contexto de los refugiados. La reducción de los síntomas se observó en algunos de los estudios.
26	Dekel R, Goldblatt H (2008)	Revisar la transmisión intergeneracional del TEPT de padres a hijos en familias de veteranos de guerra	Se ha encontrado que algunas características violentas derivadas del padre hacia los niños producto de algún síntoma de TEPT pueden afectar o tener consecuencias para el desarrollo de sintomatología en el niño.	Hay diferencias en el funcionamiento de la familia y la relación niño-padre si el padre desarrolló síntomas de TETP producto de la violencia o conflicto armado.
27	Qouta S, Punamäki R-L, El Sarraj E (2008)	Asociación entre la exposición a eventos traumáticos y salud mental de los niños, su desarrollo cognitivo y socioemocional	Se encontró un nivel inusualmente alto de TEPT entre los niños que experimentaron violencia y atrocidades en cuanto al impacto a largo plazo del estrés.	Los niños expuestos a traumas graves tienen problemas de concentración y baja capacidad cognitiva, por lo cual tienen dificultades para procesar nueva información y retener los viejos conocimientos.
28	Prasad AN, Prasad PL (2009)	Evaluar el impacto profundo y duradero en el funcionamiento emocional, cognitivo, conductual y fisiológico de un individuo	El trauma durante la infancia puede tener un efecto devastador sobre el desarrollo del cerebro y todas las funciones mediadas por este órgano complejo.	Los niños que viven en zonas de guerra pueden expresar angustia por varios eventos traumáticos a través de problemas emocionales tales como TEPT, trastornos disociativos, ansiedad y consumo de sustancias psicoactivas.
29	Jordans MJD, Tol WA, Komproe IH, De Jong JVTM (2009)	Evaluar intervenciones de bienestar psicosocial y salud mental de niños afectados por violencia en países de bajos y medianos ingresos	Los estudios de efectos están sesgados geográfica mente y apuntan a los síntomas del TEPT. Consenso a favor de enfoques basados en la comunidad multinivel, adaptaciones culturales y evaluaciones	Los modelos de rehabilitación basados en la comunidad tienen un costo más bajo. Hay una brecha entre las necesidades de salud mental y la disponibilidad de intervenciones comprobadas.
30	Persson TJ, Rousseau C (2009)	Describir las intervenciones escolares específicas y generales para niños y adolescentes en países expuestos a la guerra	Los programas de acompañamiento psicológico en niños, niñas y adolescentes en los colegios reducen los síntomas de TEPT, mantienen la esperanza, reducen la depresión y los síntomas de intrusión.	Las intervenciones de salud mental en las escuelas pueden facilitar la detección y el tratamiento de niños refugiados cuya salud mental y discapacidad funcional requieren mayor atención.
31	Sullivan PM (2009)	Revisar investigaciones sobre los tipos de victimización por violencia abordados entre niños con discapacidades	La identificación del perpetrador de la violencia también tiene relevancia para los modos y objetivos de intervención de los efectos psicológicos y de salud mental de la victimización.	Las niñas, niños y jóvenes con algún tipo de discapacidad tienen un mayor riesgo de ser víctimas de algún tipo de violencia durante su infancia, años escolares o adolescencia.
32	Attanayake V, McKay R, Joffres M, Singh S, Burkle F, Mills E (2009)	Determinar la prevalencia de trastornos mentales en niños afectados por la guerra	La infancia es un período crítico del desarrollo cognitivo, emocional y físico. Es necesaria la creación de programas para la primera infancia que garanticen el bienestar emocional, psicológico y físico de los niños en situaciones de guerra.	Los programas deben brindar apoyo a los cuidadores y abordar las necesidades básicas de los niños: la seguridad alimentaria y la nutrición, y promover el apoyo social para un desarrollo adecuado.
33	Torrado M, Camargo M, Pineda N, Bejarano D (2009)	Señalar la importancia de los impactos diferenciales del conﬂicto armado en la primera infancia	Las experiencias de la niñez que afectan sus emociones, pensamientos, comportamientos y capacidad de aprendizaje están asociadas con la forma en que los niños, perciben, asumen, asimilan, comprenden y reconstruyen tales eventos.	El conflicto afecta la construcción de la identidad y subjetividad, así como las posibilidades de reconocerse como miembro de una sociedad, de la familia, y de percibir que se es titular de derechos.
34	Steel Z, Chey T, Silove D, Marnane C, Bryant RA, van Ommeren M (2009)	Evaluar el TEPT y depresión en el campo de la salud mental de refugiados y posconflicto	La tortura surgió como el factor sustantivo más fuerte asociado con el TEPT y la exposición acumulada fue el factor sustantivo más fuerte asociado con la depresión	Aunque muchas otras variables de salud mental son importantes, la revisión se centró solo en la depresión y el TEPT, porque estas son los índices de resultado que se han estudiado suficientemente.
35	Crowley C (2009)	Evaluar las necesidades de salud mental de los niños refugiados reasentados en los Estados Unidos	Los niños refugiados exhiben una enorme cantidad de resiliencia durante todas las fases de la migración.	Los niños refugiados son un grupo de alto funcionamiento, y su rendimiento escolar y nivel de rendimiento académico a menudo no se ven afectados.
36	Peltonen K, Punamäki RL (2010)	Evaluar la efectividad de las intervenciones psicosociales entre niños traumatizados en el contexto de conflictos armados	Se encontró disminución de los síntomas de TEPT, aumento de autoestima, actitudes positivas, aceptación de resolución no violenta, rendimiento cognitivo de los niños, problemas psicológicos, síntomas de trauma y apoyo social percibido.	El metaanálisis mostró que las intervenciones basadas en las "técnicas basadas en síntomas" y "ensayos corporales" surgieron como las intervenciones más efectivas para aliviar el TEPT
37	Hernández-Barrera A, Restrepo-Espinosa M (2011)	Describir la situación actual de las condiciones de salud mental en el TEPT de la primera infancia en condiciones de desplazamiento	La exposición directa y no directa a la guerra, la clase de eventos traumáticos y la presencia de factores especiales y culturales aumentan el riesgo de problemas emocionales y comportamentales en niños.	Las experiencias traumáticas en edad temprana están asociadas a un mayor riesgo y síntomas más graves de TEPT.
38	White CJ, De Burgh HT, Fear NT, Iversen AC (2011)	Evaluar lo que se sabe sobre el impacto en los niños del despliegue parental en Irak o Afganistán	Los hijos de militares en guerra tienen la presión mucho más alta que lo normal y esto podría relacionarse con ansiedad o estrés. El primer llamado de atención para el cambio de actitud son las expresiones de estrés en niños.	Las alteraciones a nivel psicológico se evidencian en mayor medida en adolescentes que en niños por su proceso de desarrollo y las relaciones con sus figuras paternas.
39	Bronstein I, Montgomery P (2011)	Sintetizar la investigación epidemiológica sobre la salud mental de los niños refugiados que residen en los países occidentales	La edad avanzada se asoció significativamente con niveles más altos de problemas de internalización y externalización y mayores niveles de TEPT. La muerte violenta de un familiar parece estar relacionada con puntuaciones más altas de TEPT.	Los niños refugiados experimentan altos niveles de angustia psicológica, depresión y problemas emocionales y conductuales.
40	Henley J, Robinson J (2011)	Resumir el conocimiento respecto a la salud mental de los niños y adolescentes refugiados	Los niños y adolescentes refugiados corren un mayor riesgo de desarrollar problemas de salud mental, incluido el estrés postraumático y una variedad de dificultades emocionales, conductuales y educativas.	Los diversos contextos en los que los niños refugiados pueden entrar en contacto con los médicos de salud mental incluyen los servicios de salud escolar, servicios de protección infantil y hospitales.
41	Yahav R (2011)	Revisar el impacto de la exposición directa e indirecta a la guerra y el terrorismo en la salud mental de los niños	Los niños pueden sufrir de trastorno por estrés postraumático (TEPT), así como otros tipos de psicopatología que no son específicos de la experiencia de trauma, como ansiedad y depresión generales.	La exposición directa e indirecta al conflicto pone al niño en riesgo de problemas de salud mental a corto y largo plazo. La duración y el alcance de la exposición determinan la intensidad de las respuestas de los niños.
42	Montgomery E (2011)	Revisar la evidencia de trauma y salud mental relacionada con el exilio en jóvenes refugiados de Medio Oriente	Los niños presentan principalmente ansiedad, el 67 % fue clasificado con ansiedad clínica, el 34 % estaba triste o deprimido, y 238 de los 311 niños (77 %) sufrían al menos una de estas condiciones.	El tipo de evento traumático o estresante puede ser un indicador de interrupción en los sistemas ecológicos mutuos, revelando que cualquiera de esos puede afectar sistemas adaptativos fundamentales.
43	Bohleber W (2012)	Evaluar los efectos del estrés traumático en el niño en desarrollo, discutir el papel de la memoria y el recuerdo al aceptar el pasado	La realidad traumática abruma la defensa del ego y sus recursos adaptativos, trayendo siempre una sensación de impotencia, ansiedad automática y una regresión a las funciones primitivas del ego.	Las funciones integradoras de la memoria se cierran y surge un estado disociado del yo, combinado con una despersonalización y una “desrealización”
44	Werner EE (2012)	Revisar y reflexionar sobre estudios que han explorado los efectos de la guerra en los niños de todo el mundo	En relación con los efectos de los escenarios de conflicto se identifican síntomas de trastorno de estrés postraumático (TEPT), depresión y trastornos de ansiedad, y discapacidad cognitiva.	Cuanto más reciente es la exposición a la guerra, y cuanto mayor es el niño, mayor es la probabilidad de presentar síntomas de trastorno de estrés postraumático.
45	Masten AS, Narayan AJ (2012)	Analizar los modelos plausibles para la inclusión biológica del estrés extremo	Las habilidades cognitivas (inteligencia general y flexibilidad cognitiva) y las habilidades de autorregulación están ampliamente implicadas como factores protectores para los niños en una variedad de circunstancias peligrosas.	La inclusión biológica del estrés extremo en el desarrollo humano provee explicaciones cada vez más plausibles de procesos mediadores que explican sus efectos en la salud y el bienestar.
46	Drury J, Williams R. (2012)	Evaluar aspectos psicosociales de respuestas de niños y jóvenes a su exposición a la guerra, la violencia colectiva y el terrorismo	Diferentes tipos de alteraciones psicosociales como acercamiento a la muerte, soñar con la muerte de otros, etc. Se ha evidenciado que la separación de padres o de su cultura influye en la aparición de alteraciones mentales.	El daño en la salud mental de los niños está relacionado directamente con la pérdida de soporte parental.
47	Dimitry L (2012)	Revisar la salud mental de los niños y adolescentes que viven en áreas de conflicto armado en el Medio Oriente	En Israel la prevalencia del síndrome de estrés postraumático fue de 5-8 %, el 25-35 % reportó depresión leve, el 3,3 %, depresión mayor, el 3 %, déficit de atención e hiperactividad, el 2,5 %, fobias específicas, el 1,8 %, desorden desafiante, el 1,4 %, ansiedad y el 1,2 %, desorden compulsivo-obsesivo.	Entre los factores de riesgo hay asociaciones positivas de los síntomas de síndrome de estrés postraumático, depresión, ansiedad, problemas de conducta, de atención y emocionales con las exposiciones a acciones violentas.
48	Reed R V, Fazel M, Jones L, Panter-Brick C, Stein A (2012)	Evaluar factores de protección y riesgo para la salud mental de niños y adolescentes	Los niños desplazados pueden tener más problemas psicológicos que sus pares no reubicados, a pesar de cierta exposición a conflictos compartidos.	Los problemas de salud mental no son el resultado de una sola causa, sino de cadenas causales complejas. Comprender cómo interactúan los diferentes factores requiere una atención cuidadosa.
49	Fazel M, Reed R V, Panter- Brick C, Stein A (2012)	Sintetizar la evidencia de factores de riesgo y protección para la salud mental en niños y adolescentes que son desplazados por la fuerza	La experiencia de eventos adversos se asocia con una mayor probabilidad de trastornos psicológicos, el grado de TEPT se asoció con experiencias personales de eventos traumáticos, especialmente aquellos que ocurren cuando se está lejos de casa.	La exposición a la violencia fue fuertemente predictiva de trastornos psicológicos. Las adversidades acumulativas empeoran los resultados de salud, con mayores efectos que cualquier factor solo.
50	Betancourt TS, Meyers-Ohki SE, Charrow AP, Tol WA (2013)	Describir intervenciones psicosociales y de salud mental para abordar las necesidades de salud mental de los niños afectados por conflictos	Entre los niños y jóvenes expuestos a conflictos, los resultados adversos de salud mental provocados por la exposición a eventos horribles se ven agravados por el daño relacionado con la guerra a los sistemas de apoyo extendidos (familiares, sociales, económicos, políticos).	En las estrategias interdisciplinarias deben seleccionarse los sectores de salud, negocios y administración para guiar los futuros análisis de políticas y la evaluación de su implementación en la salud mental global.
51	Forman-Hoffman VL, Zolotor AJ, McKeeman JL, Blanco R, Knauer SR, Lloyd SW, et al. (2013)	Evaluar la efectividad de las intervenciones dirigidas al estrés traumático entre los niños expuestos a eventos traumáticos	Algunas intervenciones de psicoterapia dirigidas a niños expuestos a eventos traumáticos parecen prometedoras en función de la magnitud y precisión de los efectos.	Estas intervenciones fueron tratamientos escolares con elementos de terapia cognitivoconductual.
52	Betancourt T S, McBain R, Newnham EA, Brennan RT (2013)	Investigar factores de riesgo y protección asociados con el cambio de síntomas de TEPT entre los niños exsoldados en Sierra Leona	Síntomas relacionados con el trastorno por estrés postraumático (TEPT), trastorno de depresión, trastorno de ansiedad, y discapacidad funcional	La aceptación familiar, el apoyo social y las oportunidades educativas y económicas se asociaron con un mejor ajuste psicosocial.
53	Rousseau C, Jamil U, Bhui K, Boudjarane M (2013)	Evaluar el efecto del 11/09 y la guerra contra el terrorismo en niños y jóvenes mayoritarios y minoritarios en USA	Las consecuencias en preescolares y niños dependen de las reacciones familiares y la habilidad emocional del cuidador principal para afrontar lo sucedido	La familia tiene un rol protector en la modulación y los efectos del terrorismo en la reacción de los niños y un papel importante en las asociaciones y consecuencias del evento.
54	Tol WA, Song S, Jordans MJD (2013)	Evaluar la resiliencia y la salud mental en niños y adolescentes víctimas de conflictos en países de bajos y medianos ingresos	Los conflictos armados afectan seriamente los determinantes sociales de la salud mental y el bienestar, incluyendo redes de cuidado familiar y comunitario, atención a necesidades básicas y educación; moralidad y espiritualidad.	El estudio de la resiliencia en niños afectados por conflictos armados puede proporcionar información crucial para el desarrollo de la salud mental y las intervenciones psicosociales.
55	Shaar KH (2013)	Evaluar prevalencia del TEPT en la población adolescente del Líbano	El TEPT en adolescentes ha sido implicado en problemas en el desarrollo, problemas mentales y escolares, abuso de drogas y alcohol, comportamiento antisocial, entre otros.	Adolescentes con TEPT mostraron mayor deterioro escolar, además de niveles más bajos de “autoeficacia”.
56	Pacione L, Measham T, Rousseau C (2013)	Describir la salud mental de los niños refugiados que han huido de la guerra	Los problemas de externalización, incluidos los comportamientos de oposición, agresivos, impulsivos, hiperactivos y antisociales, se informan en niños refugiados.	Los factores posteriores a la migración en los países de altos ingresos y las experiencias escolares positivas confieren un efecto protector, mientras que la discriminación percibida y la exposición a la violencia elevan el riesgo.
57	Agazio J, Goodman P, Padden DL (2014)	Analizar el impacto de los despliegues de apoyo en miembros de la familia del servicio activo y el personal de reserva/guardia	Los niños tienden a reaccionar con más irritabilidad y son poco comunicativos; también se han identificado alteraciones de sueño, alimentación y periodos de sensibilidad. Los adolescentes toman aún más responsabilidades del hogar resultando en una mayor interrupción de sus rutinas normales.	Los niños escolares pueden tener más problemas en el desarrollo educativo o dificultades de aprendizaje producto de las separaciones con la figura parental, el contexto de guerra, las relaciones de responsabilidad que asume y la influencia de los padres.
58	Fu C, Underwood C (2015)	Revisar los programas de intervención dirigidos a niños y adolescentes expuestos a desastres o conflictos	La mala salud mental en la infancia se asocia con una mayor prevalencia de mala salud física, conductas de riesgo y lesiones no intencionales, suicidio y menor calidad de vida.	Las evaluaciones de las intervenciones de salud mental y apoyo psicosocial dirigidas a niños y adolescentes son relativamente limitadas.
59	Foster H, Brooks-Gunn J (2015)	Revisar las consecuencias para la salud mental de la exposición de los niños a la violencia comunitaria y de guerra	El estigma en la comunidad y la seguridad percibida transmiten efectos a los niños y la exposición a la violencia de guerra afecta su salud. Los recursos de protección incluyen la aceptación de la familia y la comunidad, el apoyo escolar y la participación en programas extracurriculares.	El proceso de estrés comienza fundamentalmente con las condiciones sociales y se articula con factores mediadores y moderadores para discernir cómo la violencia afecta la salud mental.
60	Rosshandler Y, Hall BJ, Canetti D (2016)	Examinar los factores de riesgo psicosocial para el TEPT organizado en un marco ecológico	La exposición crónica a la violencia política puede interrumpir el desarrollo normativo y conducir a consecuencias neurocognitivas, psicosociales y psiquiátricas duraderas, por lo que la exposición es perjudicial para los adolescentes.	La violencia política que tuvo lugar durante este período tuvo profundos efectos psicológicos en los adolescentes, con consecuencias negativas a nivel comunitario, individual y micro y macroecológico.
61	Miller KE, Jordans MJD (2016)	Establecer las razones para la priorización de intervenciones centradas en el niño en comunidades de conflicto	La exclusión social de niños excombatientes y jóvenes sobrevivientes de violencia sexual aumenta, y las oportunidades de juego y amistad disminuyen a medida que las familias son desplazadas y los espacios seguros desaparecen.	Las intervenciones grupales basadas en la escuela tienen potencial para aumentar el apoyo de los compañeros y reducir el aislamiento social entre los niños.
62	Slone M, Mann S (2016)	Examinar los efectos de la exposición a la guerra y el conflicto en niños pequeños	Se evidenció alta prevalencia de stress postraumático en niños expuestos en el conflicto palestino-israelí. Se reporta el incremento de la ansiedad en niños reflejados en actitudes como nerviosismo, reacciones fuertes y miedos inexplicables.	Se evidenció que el funcionamiento de la familia y de los padres es un factor promotor de la estabilidad del niño, pues reduce la presentación de síntomas relacionados con el conflicto o la guerra.
63	Jordans MJD, Pigott H, Tol WA (2016)	Proporcionar una actualización integral en las intervenciones para niños afectados por conflictos armados	La intervención implementada por la comunidad utilizando la terapia de exposición narrativa demostró resultados positivos en niños excombatiente, incluso después de un período de seguimiento de 1 año.	Invitar a líderes de la comunidad a que ayuden a diseñar programas para darles voz en el diseño de las intervenciones que coincidan con las necesidades de las comunidades.
64	Sleijpen M, Boeije HR, Kleber RJ, Mooren T (2016)	Revisar estudios sobre las formas en que los jóvenes refugiados enfrentan la adversidad para abordar sus fuentes de resiliencia	Las fuentes de resiliencia ayudaron a lidiar con recuerdos traumáticos y a hacer frente a sus vidas turbulentas: apoyo social, estrategias de aculturación, educación, religión, evitación y esperanza.	Es necesario enfatizar en la importancia de considerar el contexto social, político y cultural más amplio en el que está incrustada la vida de los jóvenes refugiados, con el fin de no enfatizar excesivamente el TEPT en jóvenes refugiados.
65	Hassan G, Ventevogel P, Jefee-Bahloul H, Barkil-Oteo A, Kirmayer LJ (2016)	Proporcionar información sobre aspectos culturales de la salud mental y el bienestar psicosocial relevantes para la atención y el apoyo a los sirios afectados por la crisis	Los estudios de niños refugiados sirios han documentado una amplia gama de problemas psicosociales que incluyen: temores y ansiedad persistente, dificultades para dormir, tristeza, dolor y depresión, agresión o rabietas, nerviosismo, hiperactividad y tensión, problemas del habla o mutismo, entre otros.	Las intervenciones psicosociales deben estar enmarcadas en una atención mental y en la posibilidad de proveer diferentes oportunidades educativas, laborales, ocupacionales y estar enmarcadas en centros comunitarios, puntos de reunión popular, centros deportivos, culturales, entre otros.
66	Neal S, Stone N, Ingham R (2016)	Describir los resultados de salud sexual y reproductiva entre mujeres jóvenes afectadas por la exposición a conflictos armados	Ante el conflicto armado en Uganda hubo un cambio completo en los valores culturales, incrementó de las relaciones sexuales y el matrimonio transaccional, la violencia y los comportamientos sexuales de alto riesgo, y múltiples parejas.	El aumento del matrimonio precoz en Siria parece haber ocurrido como una convergencia de preocupaciones sobre el honor y la seguridad de las mujeres solteras en tiempos de agitación y violencia.
67	Saltzman LY, Solomyak L, Pat-Horenczyk R (2017)	Revisar la literatura reciente sobre las necesidades de salud mental de los jóvenes en el contexto de la guerra y el terrorismo	Existen varios enfoques para tratar a las personas y familias expuestas a eventos potencialmente traumáticos: (1) terapia cognitiva conductual, (2) desensibilización y reprocesamiento del movimiento ocular, (3) terapia dialéctica comportamental, (4) terapias alternativas.	Se deben implementar programas a partir del uso del internet como medio de intervenir a jóvenes de todas partes, usando una variedad de plataformas multimedia.
68	Stough LM, Ducy EM, Kang D (2017)	Revisar la literatura sobre factores psicosociales relacionados con los niños con discapacidades en el contexto de desastres o terrorismo	Las personas con discapacidad intelectual también tienen un mayor riesgo de TEPT debido a la exposición previa a traumas de varios tipos.	Los niños con discapacidades experimentan una exposición amplificada a los conflictos debido al aumento de las vulnerabilidades psicológicas, físicas y educativas.
69	Anagnostopoulos DC, Giannakopoulos G, Christodoulou NG (2017)	Discutir los contextos que configuran las crisis económica y migratoria y sugerir posibles efectos de esta intersección sobre los riesgos para la salud mental	Consecuencias perjudiciales de estas crisis en el bienestar psicológico, la depresión, los trastornos de ansiedad, el insomnio, el abuso del alcohol y el comportamiento suicida. Los defectos sociales externos interactúan con déficits individuales internos y comportamientos de actuación.	Se encontraron condiciones de trabajo precarias, desigualdades, inseguridad, falta de conexión social, pérdida de apoyo social, e inestabilidad de la vivienda.
70	Brown FL, de Graaff AM, Annan J, Betancourt TS (2017)	Evaluar intervenciones psicosociales para niños y jóvenes afectados por conflictos que viven en países de bajos y medianos ingresos.	Se identificaron elementos comunes en intervenciones efectivas para niños afectados por conflictos: promoción del acceso, psicoeducación, construcción de ideas, técnicas de construcción de una buena relación, estrategias cognitivas, uso de narrativas, técnicas de exposición y prevención de recaídas.	Existen varias consideraciones únicas al desarrollar intervenciones para niños en entornos afectados por conflictos, donde la disponibilidad de especialistas para la implementación, capacitación y supervisión es extremadamente limitada.
71	Kadir A, Shenoda S, Goldhagen J (2018)	Revisar evidencia sobre los efectos del conflicto armado en los niños apoyados en la Declaración de política sobre conflictos armados	Los niños afectados por la guerra tienen una mayor prevalencia de TEPT, depresión, ansiedad y quejas conductuales y psicosomáticas. Los que se asociaron con grupos armados experimentan riesgos particulares de salud física y del desarrollo mental; barreras en el acceso a los servicios de salud y obstáculos importantes para la reintegración social.	El conflicto armado es un determinante social descuidado de la salud infantil, y los efectos agudos y crónicos del conflicto armado en la salud infantil y el bienestar se encuentran entre las mayores violaciones de los derechos del niño en el siglo 21.
72	Fajardo-Mayo MA, Ramírez- Lozano MP, Suescún V, Isabel M, Ospina-Alvarado MC (2018)	Revisar estudios sobre estado del arte del conocimiento producido en Colombia entre 2002 y 2012 sobre la identidad y subjetividad de niños y niñas en torno a la paz y la democracia en contextos de conflicto armado	Existe afectación en la salud mental de niños y niñas que han sufrido desplazamiento forzado o reclutamiento que les dificulta o impide su adaptación a nuevos entornos socioculturales o a la vida civil. Los niños y las niñas, a pesar de haber vivido de manera directa o indirecta el contexto de conflicto armado, tienen en sí mismos y en sus relaciones, potencias y recursos que aportan a la construcción de subjetividades alternativas a la violencia y a la victimización.	Se considera a los niños y niñas con capacidades diversas de acción, orientadas por sus propias capacidades de sentir, expresar, crear y actuar, en diálogo con lo que sucede en sus relaciones en diferentes contextos como la vida en la escuela, en la familia y en la comunidad.

TEPT: trastorno de estrés postraumático

### Principales consecuencias en la salud mental

Según los estudios revisados, las consecuencias negativas predominantes en la salud mental en el periodo previo al conflicto, el conflicto y el posconflicto, se enmarcaron en el espectro de la depresión, la ansiedad y el estrés postraumático.

En el periodo previo a la consolidación del conflicto, se reportaron con mayor frecuencia reacciones somáticas, como cefalea ^25^^-^^29^ y dolor físico y emocional ^26^^,^^27^^,^^30^^,^^31^. Asimismo, se mencionaron fenómenos relacionados con la desintegración familiar progresiva ^32^ y el estrés previo a la migración, con un incremento de los síntomas de dolor y desesperanza ante la necesidad de salir de los territorios, el miedo de ser atacados y enfrentarse a lo desconocido ^30^^,^^33^^,^^34^, reacciones que se veían intensificadas en el inicio del conflicto ^35^.

Durante el conflicto propiamente dicho, se describen reacciones agudas y crónicas en función de su duración. Entre las agudas, predominaron los problemas en la relación con los padres y la disminución de las actividades sociales ^27^^,^^36^^,^^37^. Otras reacciones agudas señaladas fueron miedo, terror o nerviosismo ^38^^-^^42^, retroceso en habilidades adquiridas (enuresis) e hiperactividad o déficit de atención ^23^^,^^42^^-^^44^, así como tristeza, ira, vergüenza y culpa ^41^^-^^43^^,^^45^^-^^47^. En cuanto a las consecuencias que se extienden en el tiempo, diferentes investigaciones reportan conductas agresivas ^40^^-^^42^^,^^48^^-^^50^^)^ que, conjuntamente con la hostilidad y otras formas de violencia, tienden a incrementarse ^51^^,^^52^. Incluso con supervisión de los padres, la conducta agresiva permanece años después de finalizado el conflicto ^8^^,^^33^^,^^42^.

Los síntomas del espectro de la depresión se superponen con las manifestaciones del espectro de ansiedad y alteraciones de la conducta. Se describen reacciones emocionales de irritabilidad y llanto, pensamientos de miseria y soledad, así como conductas oposicionistas y antisociales ^23^^,^^53^^-^^58^. En diversos estudios, la tendencia a presentar síntomas depresivos fue mayor en quienes experimentaron la separación forzada de sus padres ^33^^,^^58^.

En cuanto al espectro de ansiedad, los síntomas eran inespecíficos y algunos de ellos se interrelacionaban con los propios del estrés postraumático, siendo frecuentes los temores de separación, alteraciones de sueño, quejas somáticas e hipersensibilidad ^56^^,^^57^^,^^59^^-^^63^. En el estudio de Montgomery, *et al.,* los niños refugiados de Oriente Medio durante 1991 y 1992 mostraban diversos síntomas psicológicos ^64^: el 67 % fueron clasificados con ansiedad clínica y el 34% con depresión; en tanto que 238 de los 311 niños bajo tratamiento (el 77 %) sufrieron por lo menos una de estas condiciones ^64^.

Los síntomas del espectro postraumático (TEPT) se describen desde temprana edad. En niños entre los 2 y los 7 años, se mencionan temores secundarios a la separación familiar, reacción exagerada al ruido, estados nerviosos, agresividad y agitación, llanto excesivo, pesadillas o activación ansiosa ^17^. En la niñez media y la adolescencia, otros estudios reportaron conductas de evitación, pérdida del apetito y de energía, y sentimientos de culpa ^65^^-^^71^. Berman, *et al.,* encontraron una correlación entre los síntomas, el número y tipo de exposiciones traumáticas ^33^. Además, varios autores coincidieron en que el estrés postraumático se relacionaba estrechamente con la alteración del proceso cognitivo-emocional de la experiencia dolorosa y la poca capacidad para asimilar el trauma ^33^^,^^69^^,^^72^^,^^73^.

Por otra parte, los estudios evidenciaron que las particularidades de cada conflicto y la exposición específica de los niños o los adolescentes a ciertos actos de guerra determinaban la mayor o menor emergencia de síntomas enmarcados en el espectro de síndromes o trastornos mentales. Así, en el conflicto palestino-israelí, la prevalencia de estrés postraumático en niños y adolescentes varió entre 23 y 70 %, la ansiedad, entre 40 y 100 %, y la depresión aquejaba al 11,3 % de esta población en Palestina. En Israel, la prevalencia de estrés postraumático fue de 5 a 8 %, la de depresión leve, de 25 a 35 %, y la de depresión mayor, de 3,3 % ^17^. En otros estudios relacionados la segunda *intifada* o levantamiento de la mezquita Al-Aqsa, en Israel y Palestina, durante y después del conflicto como tal, se reportó una prevalencia de TEPT de 58 a 65 % ^42^. Por otro lado, entre los niños refugiados en países europeos provenientes de Somalia, Sudán, Etiopía, Kosovo, Bosnia y Albania, la prevalencia osciló entre 19 y 54 % ^55^. En otro estudio de niños y adolescentes refugiados en Suecia procedentes de Israel, el 19 % presentaba algún tipo de síntoma o afectación postraumática ^16^.

En cuanto al curso clínico, los estudios consultados evidenciaron variaciones en la psicopatología a lo largo del tiempo. Algunos indicaron una aparición tardía de los síntomas de estrés postraumático y, una disminución, cuando las situaciones contextuales cambiaron, con un margen de tiempo indeterminado ^35^^,^^42^^,^^58^^,^^71^. Según Barenbaum, *et al.,* los síntomas diferían según la duración, la exposición o el recrudecimiento del conflicto. Es así como solo el 22 % de los infantes israelíes presentó algún síntoma posterior a un ataque con misiles, en tanto que, en el conflicto con Kuwait después de cinco meses de conflicto, el 70 % de los niños y niñas todavía manifestaba estos síntomas ^74^. Otros datos adicionales sobre conflictos armados en diferentes contextos geopolíticos ilustran las variaciones en el inicio y la duración de los síntomas postraumáticos y afectivos.

Después de la *intifada* de Al-Aqsa en Israel y Palestina en el 2000, se reportó el TEPT como un efecto a largo plazo, especialmente en adolescentes, que disminuyó solo con el tiempo ^42^. Por otra parte, el tiempo de disminución de los síntomas en el primer ataque terrorista al *World Trade Center* en el 2009 fue de 9 meses ^45^; en el segundo ataque del 2011, disminuyeron 6 meses después, el 10,5 % de los infantes desarrollaron estrés postraumático y menos del 25 %, alguna alteración mental ^45^^,^^60^. En el 2010, una vez finalizó el conflicto armado en Gulu, norte de Uganda, 205 adolescentes presentaron diferentes tipos de alteraciones cognitivas: pensamiento recurrente de acercamiento a la muerte, soñar con la muerte de otros, inseguridad frente a los ataques y represalias, entre otros. El 57 % de ellos reportaba síntomas incluso cuatro años después de finalizado el conflicto ^58^. Entre los infantes refugiados, se reportaron tasas de ansiedad del 35 al 87 % cuando ya habían sido alejados del conflicto ^73^. Se observó que la prevalencia de síntomas aumentaba si las experiencias se relacionaban con hechos de violencia contra sus padres o familias ^58^^,^^75^.

Por último, en el posconflicto inmediato, se ha podido establecer que el sufrimiento producto de la guerra no se detiene al trasladarse a un sitio más favorable ^65^ y que, además, hay una probable transmisión de padres a hijos ^25^^,^^48^^,^^65^^,^^76^^,^^77^. En ese sentido, en el estudio de van Ijzendoorn, *et al.,* en los descendientes infantes y adolescentes de personas que participaron en la II Guerra Mundial, se encontró una menor adaptación al trauma, y psicopatologías producto de las relaciones con los padres y con factores histórico-contextuales ^48^. La persistencia de los efectos negativos se observa asimismo en un estudio en África en el cual se evidenció que, una vez cesaron los conflictos, los excombatientes, especialmente adolescentes, eran más proclives a experiencias extremas de hostilidad y ansiedad, y mostraban un deterioro progresivo de la resiliencia ^58^.

En consonancia con las variaciones en el curso clínico, en diferentes estudios consultados se detectaron procesos de afrontamiento, como resolución de problemas, descarga emocional y búsqueda de apoyo social, relacionados con la disminución de la sintomatología y la resiliencia, definida esta como el mecanismo para amortiguar los efectos del trauma mediante el sentido de agencia, el ajuste emocional, la inteligencia social y la empatía ^35^^,^^49^^,^^59^^,^^78^^,^^79^. Los factores protectores sumados a la resiliencia, como la disposición individual y de la familia (apoyo y acompañamiento) y el soporte social (pares, escuela y comunidad), facilitaron a los infantes y adolescentes sobreponerse rápidamente ^35^^,^^49^.

En el posconflicto también se ha observado que, conforme pasa el tiempo, la población tiende a experimentar estrés por los prejuicios sociales derivados del conflicto armado ^76^. En algunos estudios esto se correlacionó con adversidades adicionales producto de un ambiente inadecuado y propiciador de otros eventos traumáticos con la consecuente disminución de la capacidad para mitigarlos ^65^^,^^76^. Davis, *et al.,* mencionan que los niños inmersos en un ambiente protector, especialmente quienes logran reestablecer lazos afectivos con algún miembro de su familia, tienen mejores resultados en el tratamiento de las consecuencias mentales y sociales ^69^.

### Consecuencias psicosociales

Entre las consecuencias psicosociales, las dificultades en la construcción de la identidad y subjetividad fueron las más relevantes en los estudios revisados, por su papel en el proceso de identificación como miembro de una sociedad y el cumplimiento de una serie de normas sociales ^39^^,^^49^^,^^56^.

En diversos estudios se evidenció una mayor vulnerabilidad en niños y adolescentes con exposición a la explotación sexual y el consumo de sustancias psicoactivas ^11^^,^^38^^,^^49^^,^^78^^,^^80^. Esta vulnerabilidad se asoció con la tendencia a asumir roles adultos prematuros de tipo laboral, administración del dinero y adopción de decisiones ^26^^,^^32^^,^^56^^,^^75^^,^^81^. Dichas actividades se asociaron con particularidades del desarrollo de la moralidad en niños de 6 a 11 años, específicamente con la posibilidad de tomar decisiones que justifiquen o no el conflicto armado ^9^^,^^21^^,^^74^. Además, se plantea que una moralidad favorable a los conflictos armados entraña el peligro real de que estas personas conformen o se unan a pandillas o grupos delincuenciales, con el fin de justificar comportamientos violentos ^49^^,^^77^.

Otros resultados psicosociales relevantes son los asociados con la educación y los procesos cognitivos. En el aspecto educativo, se encontraron problemáticas en el rendimiento escolar y dificultades de aprendizaje ^47^^,^^51^^,^^60^^,^^61^^,^^80^. Entre las consecuencias graves de tipo cognitivo, se resaltan las relacionadas con la malnutrición ^38^^,^^44^^,^^47^^,^^49^^,^^61^^,^^82^, y el compromiso de las funciones cognitivas básicas (memoria, atención y concentración, sensación y percepción) y las superiores (pensamiento, conciencia y razonamiento) ^42^^,^^60^^,^^61^^,^^80^^,^^82^.

Por último, se encontró que el entorno social y familiar tuvo un efecto mediador en el proceso de afrontamiento de las experiencias traumáticas en los infantes, en su capacidad de sobreponerse a ellas y en algunos resultados psicosociales ^31^^-^^33^^,^^57^^,^^60^^,^^68^^,^^83^. También, se señalaron adversidades como la pobreza, la separación familiar y las actitudes de racismo, las cuales favorecieron la permanencia de la sintomatología ^33^^,^^40^^,^^52^. Por otro lado, los problemas relativos al apoyo y la estructura social parecen influir en el aumento de los intentos de suicidio, los comportamientos perturbadores y los episodios disociativos en adolescentes ^31^^,^^40^. Anagnostopoulos, *et al.,* señalaron que el desarrollo personal y social se veía afectado por la exposición a los conflictos armados y a inseguridades internas que obstaculizan la conexión social ^40^. Por último, Neal, *et al.,* registraron un aumento de los matrimonios precoces producto del conflicto armado, así como de prácticas culturales en respuesta al colapso de la cohesión comunitaria ^62^.

## Discusión

Esta revisión proporciona una síntesis de la evidencia en torno a las consecuencias de los conflictos armados en la salud mental de la población infantil y adolescente a lo largo de sus distintos momentos, así como de procesos y contextos mediadores de los efectos negativos y mecanismos de resiliencia que revisten importancia en el campo de las intervenciones individuales y colectivas en este tipo de población.

Los estudios incluidos en esta revisión coinciden en que las consecuencias en la salud mental se derivan de la interacción de un conjunto de variables y afectaciones que pueden enmarcarse en un síndrome o trastorno específico según las alteraciones afectivas, cognitivas o conductuales predominantes en un momento dado.

Cabe resaltar que predominó la descripción de consecuencias negativas del espectro de la depresión, la ansiedad y los trastornos postraumáticos a lo largo de los distintos momentos del periodo previo al conflicto, el conflicto como tal y el posconflicto, independientemente de que los estudios consultados se llevaron a cabo en contextos y temporalidades tan disímiles como los conflictos armados vividos en Oriente Medio durante la década de 1990 ^69^, el conflicto palestino-israelí ^16^, los conflictos de Somalia, Sudán, Etiopía, Kosovo, Bosnia y Albania ^55^, el conflicto de Kuwait ^74^, la Segunda Guerra Mundial ^84^, o el de Sierra Leona ^85^, entre otros, pues a pesar de las particularidades de cada conflicto y la exposición específica de los niños o adolescentes a ciertos actos de guerra, son determinantes de la mayor o menor incidencia de cierto tipo de sintomatologías.

Hoy se sabe que la intensidad, la brusquedad y la duración de los conflictos armados están asociadas con la aparición de la psicopatología ^14^^,^^25^^,^^46^. Estos hallazgos son consistentes con revisiones previas que han señalado la relación entre una exposición mayor a eventos traumáticos durante las etapas tempranas del ciclo vital y la presencia de síntomas más graves, especialmente de tipo afectivo ^86^^,^^87^.

La superposición de espectros sintomáticos de condiciones de salud mental evidenciada en esta revisión, concuerda con la amplitud y la diversidad de los problemas de salud mental en infantes y adolescentes dadas las particularidades evolutivas de esta población, las cuales pueden abordarse a partir de su manifestación externa (oposicionismo, agresividad, hiperactividad, consumo de sustancias psicoactivas, comportamiento delictivo) o interna (ausencia de motivación, tristeza, ideación suicida, desesperanza, temores) ^87^.

En cuanto al momento temporal del conflicto, las primeras afectaciones se relacionan principalmente con la salud física, tanto por las limitaciones funcionales que resultan de la incapacidad de cuidarse por sí mismos ^87^, como por la somatización de la ansiedad asociada con la percepción de amenaza y la exposición a escenarios de violencia ^88^, en tanto que las consecuencias a largo plazo, una vez finalizado el conflicto, se relacionan más con depresión, dificultades de regulación emocional y problemas de conducta ^23^^,^^87^. Los síntomas agudos del espectro postraumático y de ansiedad descritos en los estudios revisados, se relacionaron con alteraciones en la reactividad al estrés mediadas por reacciones hormonales del eje hipotalámico-hipofisario-suprarrenal, evaluadas mediante la medición del cortisol basal y otras hormonas ^89^. Estas alteraciones, documentadas en una cohorte de infantes expuestos a experiencias de guerra y en adultos separados de sus padres en la infancia durante la Segunda Guerra Mundial, podrían explicar la asociación entre el desarrollo de problemas afectivos y las experiencias de separación forzada de los padres que se reporta en los estudios incluidos en la presente revisión ^90^^,^^91^.

Ante la heterogeneidad de problemas de salud mental y biopsicosocial encontrada, vale la pena profundizar en otros factores potencialmente causales, como el grado de participación y el tipo de victimización en el conflicto armado, así como la pérdida de la estabilidad de la red comunitaria ^46^. En este sentido, en la presente revisión se evidencian las complejidades de infantes y adolescentes refugiados, un grupo con mayor riesgo de afectación debido a los extremos de estrés a los cuales se enfrentan en todos los momentos de un conflicto armado ^41^^,^^75^, así como la necesidad de abandonar el país de origen, desplazarse a otro y tener que adaptarse en él ^30^^,^^34^^,^^75^.

En cuanto a los niños y adolescentes combatientes, se destacan las condiciones de explotación y adoctrinamiento basadas en dimensiones económicas, sociales y políticas ^23^^,^^49^^,^^58^^,^^74^. Las situaciones de violencia sexual y otras consecuencias negativas en salud sexual y reproductiva, se documentan en los estudios sobre conflictos armados en algunas regiones geográficas, los cuales señalan cómo las afectaciones en la salud mental de los cuidadores aumentan el riesgo de este tipo de victimización que, como es sabido, también incrementa el riesgo de desarrollar psicopatologías a corto y largo plazo ^89^, sin contar con las consecuencias de estas experiencias en el desarrollo psicosocial, moral y en la construcción de identidad ^27^^,^^29^^,^^49^.

En Colombia, un estudio del Instituto Colombiano de Bienestar Familiar (ICBF) sobre el estado psicosocial de niños y adolescentes afectados por diferentes hechos de violencia en el marco del conflicto armado, guarda relación con los hallazgos de la presente revisión, ya que, entre los niños desmovilizados o excombatientes, se encontró una afectación significativa en la autoestima y el bienestar subjetivo acompañada por la incapacidad de sentir alegría y felicidad, la aparición de conductas agresivas y un comportamiento transgresor de la normatividad, en tanto que en los desplazados, es decir, quienes han sido obligados a dejar su hogar y su red de apoyo debido a los enfrentamientos armados, se encontraron problemas sociales, preocupaciones por la muerte y dificultad en el manejo de las emociones ^88^.

Una limitación de este estudio fue la amplitud y la cantidad de la información revisada. Sin embargo, constituye un aporte relevante y actualizado que ilustra la necesidad de sintetizar los resultados obtenidos en diferentes estudios a lo largo del tiempo. Otra limitación es que la mayoría de las revisiones incluidas eran de tipo narrativo, lo que resta validez a los hallazgos. Los estudios primarios de las revisiones sistemáticas fueron, en un porcentaje importante, series de casos o estudios de corte transversal, diseños que no permiten establecer una relación de causalidad entre las experiencias de trauma directo debidas a los conflictos armados, y el desarrollo de las alteraciones psicológicas y los trastornos psiquiátricos detectados, ya que no se puede establecer la presencia de factores de riesgo previos a la exposición al conflicto armado, como malnutrición y otras complicaciones perinatales que inciden en el desarrollo cerebral. En muchos países, una proporción importante de los niños y adolescentes que se vinculan a los grupos armados en un conflicto han vivido previamente en condiciones de precariedad relacionadas con complicaciones en la salud perinatal, o han experimentado situaciones traumáticas en sus hogares ^89^. En el estudio del ICBF realizado en Colombia, se reportaron factores previos al hecho de que los victimiza, como violencia intrafamiliar, maltrato infantil, abuso sexual, desigualdad, inequidad, discriminación y exclusión social ^88^.

Es importante señalar la variedad de escalas y criterios diagnósticos empleados en la literatura revisada ^25^^,^^45^^,^^46^^,^^50^. Si bien el objetivo del presente estudio no consistía en evaluar los instrumentos y criterios diagnósticos, Jensen, *et al.,* mencionan la necesidad de contemplar la naturaleza de los factores estresantes, daños reales y particularidades de los contextos culturales y geopolíticos para poder llegar a un diagnóstico válido ^50^. Asimismo, Elbedour, *et al.,* describen la cultura como el macrosistema influyente en las respuestas individuales ^25^. Todos estos criterios y evaluaciones son muy diferentes unos de otros, lo cual aumenta la heterogeneidad de los resultados y dificulta su reproducibilidad ^15^^,^^92^.

Pese a lo anterior, se evidencia un avance en los estudios en cuanto a la evaluación de problemas de salud mental diferentes al trastorno de estrés postraumático ^15^, lo que se articula con el enfoque de otras revisiones de la literatura sobre infantes y adolescentes víctimas de conflictos armados ^23^^,^^87^^,^^93^. Sin embargo, llama la atención la escasez de estudios enfocados en otros trastornos mentales, como los del espectro psicótico ^15^^,^^31^.

En cuanto al curso clínico, la información es diversa ^8^^,^^15^^,^^75^. La duración de los síntomas es controversial, ya que depende de las oportunidades en el medio, la cultura y otros factores contextuales ^74^. En conclusión, existe una limitación para la generalización de los resultados debida a los aspectos geopolíticos de las poblaciones estudiadas. Por ejemplo, en el caso de la población de refugiados, los estudios se referían en su mayoría al conflicto armado en Oriente Medio ^34^^,^^56^^,^^73^.

Los resultados de los estudios en población infantil se relacionan estrechamente con las investigaciones sobre los efectos en la salud mental de los adultos, tanto de quienes fueron víctimas de conflictos en su vida adulta, como de quienes lo fueron durante su niñez y adolescencia, y cuyo malestar se mantiene. En este sentido, algunas revisiones del tema evidencian que los adultos expuestos a contextos de guerra durante etapas tempranas de su vida son más propensos a resolver disputas familiares con violencia, y son más proclives al abuso y la negligencia infantil ^94^. Asimismo, se observó una correlación entre el estado de salud mental de los adultos y el de niños y adolescentes, es decir, el entorno familiar y la respuesta de los padres a las situaciones traumáticas influyen de manera significativa en las estrategias de afrontamiento de sus hijos y, en general, en su bienestar ^16^.

Por otro lado, entre los resultados más relevantes se encuentran las consecuencias relacionadas con el desarrollo psicosocial, que se manifiestan en diferentes áreas, como roles forzados ^56^^,^^75^^,^^81^, bajo desempeño educativo ^51^^,^^61^^,^^80^, problemas cognitivos a largo plazo ^47^^,^^49^^,^^61^^,^^82^ e influencias de los contextos de pobreza y discriminación, entre otros ^33^^,^^40^^,^^52^. Las consecuencias psicosociales se han descrito en diversos estudios, sin embargo, no se ha examinado el cambio en el tiempo y la posible relación con afectaciones en otras áreas del desarrollo.

Una de las fortalezas de esta investigación fue la revisión extensa y detallada de los estudios en diferentes bases de datos para recoger la mayor cantidad de información posible, así como la eventual aplicación de esta síntesis en el campo de la prevención y la mitigación de las afectaciones. Del abanico de consecuencias potenciales de los conflictos armados en la salud mental de infantes y adolescentes, se desprende la necesidad de una aproximación integral, con enfoques complementarios de intervención, que confluyan en el recurso a diferentes profesiones de la salud y las ciencias sociales. Uno de los enfoques que enriquece la comprensión de los conflictos armados y sus consecuencias en la salud mental en este tipo de población, son las intervenciones socioculturales y comunitarias ^33^^,^^40^^,^^52^. En este sentido, otras investigaciones han hecho énfasis en la necesidad de que los programas de prevención y el acceso a profesionales de salud mental se gestionen según los grados y tipos de exposición al conflicto armado ^93^, y que la atención psicosocial esté focalizada en las necesidades específicas de la población teniendo en cuenta variables como etnia, edad y origen ^88^.

Debe resaltarse también que la salud mental de los cuidadores es un factor de suma importancia, debido a su relación con el posible desarrollo de problemas en infantes y adolescentes ^94^, lo que resalta el efecto sistémico de los conflictos armados en la salud mental y el desarrollo biopsicosocial, es decir, con los efectos individuales y la ruptura de las redes de apoyo social, lo que se agrava con la falta de acceso a los servicios de salud esenciales. De ello se desprende la necesidad de implementar intervenciones con un enfoque sistémico que transcienda la dimensión clínica y psicopatológica, y den respuesta a los problemas de salud individual y a la diversidad de problemas sociales, educativos y económicos causados por los conflictos armados ^95^^,^^96^.

En futuros estudios se recomienda usar el diseño longitudinal y hacer evaluaciones confiables con metodología cualitativa y cuantitativa que permitan superar las limitaciones en la recolección de la información y conocer las magnitudes del efecto en el tiempo ^8^, así como examinar la relación entre determinado tipo de experiencias de violencia y el riesgo de desarrollar afectaciones específicas en la salud mental. Dada la complejidad del tema, es necesario un mayor rigor metodológico en los estudios para obtener resultados válidos, no solo en términos de psicopatología, sino también, en los factores protectores y de resiliencia ^15^^,^^92^.

## Archivos suplementarios 

**Anexo 1:** Estrategia de búsqueda

Reporte de búsqueda electrónica No. 1	
Base de datos	MEDLINE(R) and Epub Ahead of Print, In-Process & Other Non-Indexed Citations, Daily and Versions(R)
Plataforma	Ovid
Fecha de búsqueda	01-07-2019
Rango de fecha de búsqueda	Sin restricción
Restricciones de lenguaje	Sin restricción
Otros límites	Revisiones, máxima sensibilidad
Estrategia de búsqueda (resultados)	1. exp Child/ (1834590) 2. child*.ab,ti.(1167918) 3. preschool.ab,ti. (19899) 4. adolescent.ab,ti. (91883) 5. 1 OR 2 OR 3 OR 4 (2194211) 6. exp Armed Conflicts/ (9711) 7. war.ab,ti. (30580) 8. (War and (conflict or crimen)).ab,ti. (1486) 9. 6 OR 7 OR 8 (36638) 10. 5 AND 9 (4302) 11. limit 10 to “reviews (best balance of sensitivity and specificity)” (412)
Referencias identificadas	412
Reporte de búsqueda electrónica No. 2	
Base de datos	Embase
Plataforma	Elsevier
Fecha de búsqueda	09-06-2019
Rango de fecha de búsqueda	Sin restricción
Restricciones de lenguaje	Sin restricción
Otros límites	Revisión
Estrategia de búsqueda (resultados)	1. 'child'/exp (2729007) 2. child*:ab,ti (1701964) 3. 'adolescent'/exp (1576994) 4. 'adolescence'/exp (89544) 5. teenager:ab,ti (3793) 6. 1 OR 2 OR 3 OR 4 OR 5 (3956107) 7. 'war'/exp (31055) 8. 'armed conflict*':ab,ti (1159) 9. 'crimean war':ab,ti (112) 10. 4 OR 5 OR 6 (31609) 11. 6 AND 10 (3982) 12. 8 AND 'Review'/it (360) 13.9 AND 'Review'/it AND [embase]/lim NOT ([embase]/lim AND [medline]/lim) (85)
Referencias identificadas	85
Reporte de búsqueda electrónica No. 3	
Tipo de búsqueda	Nueva
Base de datos	Cochrane Database of Systematic Reviews
Plataforma	Ovid
Fecha de búsqueda	01-07-2019
Rango de fecha de búsqueda	Sin restricción
Restricciones de lenguaje	Sin restricción
Otros límites	Revisión
Estrategia de búsqueda (resultados)	1. child.mp. [mp=title, short title, abstract, full text, keywords, caption text] (3180) 2. child*.mp. [mp=title, short title, abstract, full text, keywords, caption text] (5195) 3. preschool.mp. [mp=title, short title, abstract, full text, keywords, caption text] (576) 4. adolescent.mp. [mp=title, short title, abstract, full text, keywords, caption text] (954) 5. 1 OR 2 OR 3 OR 4 (5269) 6. armed conflicts.mp. [mp=title, short title, abstract, full text, keywords, caption text] (5) 7. war.mp. [mp=title, short title, abstract, full text, keywords, caption text] (85) 8. (War and (conflict or crimen)).mp. [mp=title, short title, abstract, full text, keywords, caption text] (22) 9.6 OR 7 OR 8 (86) 10. 5 AND 9 (60)
Referencias identificadas	60
Reporte de búsqueda electrónica No. 5	
Tipo de búsqueda	Nueva
Base de datos	LILACS
Plataforma	Biblioteca Virtual de la Salud
Fecha de búsqueda	01-07-2019
Rango de fecha de búsqueda	Sin restricción
Restricciones de lenguaje	Sin restricción
Otros límites	LILACS
	Revisión
Estrategia de búsqueda (resultados)	1. tw:(((child OR child* OR preschool OR adolescent) AND (armed conflict OR war OR (war (conflict OR crimen))) AND (review)) AND (instance:"regional") AND ( db:("LILACS"))) (12)
Referencias identificadas	12

## Anexo

**Anexo 2:** Estudios excluidos y razones

N°	Autores	Título	Razón de exclusión
1	Häfner, H., Veiel, H. O., Welz, R. (1988)	The epidemiology of suicide and attempted suicide	No enfocado en la población
2	Ursano, R. J., Holloway, H. C., Jones, D. R., Rodríguez, A. R., Belenky, G. L. (1989)	Psychiatric care in the military community: family and military stressors	No enfocado en el objetivo
3	Bar-On, D. (1990).	Children of perpetrators of the Holocaust: working through one's own moral self	No es una revisión.
4	Garbarino, J., Kostelny, K., Dubrow, N. (1991)	What children can tell us about living in danger	No enfocado en el objetivo
5	Grillo, T. (1991)	Violence among women and children	No enfocado en la población
6	Hobfoll, S. E., Spielberger, C. D., Breznitz, S., Figley, C., Folkman, S., Lepper-Green, B.,... van der Kolk, B. (1991)	War-related stress. Addressing the stress of war and other traumatic events	No enfocado en la población
7	Krell, R. (1993)	Child survivors of the Holocaust--strategies of adaptation	No es una revisión.
8	Putnam, F. W., Trickett, P. K. (1993)	Child sexual abuse: a model of chronic trauma	No enfocado en el objetivo
9	Schwarz, E. D., Perry, B. D. (1994)	The post-traumatic response in children and adolescents	No enfocado en el objetivo
10	Aboutanos, M. B., Baker, S. P. (1997)	Wartime civilian injuries: epidemiology and intervention strategies	No enfocado en el objetivo
11	Shearar, A. (1997)	Dying to go to school	No es una revisión.
12	Hodgins, S. (1998)	Epidemiological investigations of the associations between major mental disorders and crime: methodological limitations and validity of the conclusions	No enfocado en la población
13	Joshi, P. T. (1998)	Guidelines for international trauma work	No es una revisión.
14	Sack, W. H. (1998)	Multiple forms of stress in refugee and immigrant children	No es una revisión.
15	van der Hart, O., Brown, P., Graafland, M. (1999)	Trauma-induced dissociative amnesia in World War I combat soldiers.	No enfocado en la población
16	Kaminer, D., Seedat, S., Lockhat, R., Stein, D. J. (2000)	Violent trauma among child and adolescent girls: current knowledge and implications for clinicians.	Artículo no encontrado y no hay respuesta de autores.
17	Nakatani, Y. (2000).	[Dissociative disorders: from Janet to DSM-IV].	No enfocado en el objetivo ni la población
18	Ng, V., Norwood, A. (2000)	Psychological trauma, physical health and somatization.	No enfocado en el objetivo
19	Solomon, Z. (2000)	The psychological consequences of war: The Israeli experience	No es una revisión.
20	Buskila, D. (2001)	Fibromyalgia, chronic fatigue syndrome, and myofascial pain syndrome.	No enfocado en el objetivo
21	O'Shea, B. (2001)	Post-traumatic stress disorder: A review for the general psychiatrist	No enfocado en la población
22	Porter, M., Haslam, N. (2001)	Forced displacement in Yugoslavia: a meta-analysis of psychological consequences and their moderators.	No enfocado en la población
23	Dickson-Gómez, J. (2002)	The sound of barking dogs: violence and terror among Salvadoran families in the postwar.	No es una revisión.
24	Veenema, T. G., Schroeder-Bruce, K. (2002)	The aftermath of violence: children, disaster, and posttraumatic stress disorder.	No es una revisión.
25	Albertyn, R., Bickler, S. W., Van As, A. B., Millar, A. J. W., Rode, H. (2003)	The effects of war on children in Africa.	No enfocado en el o.bjetivo
26	Caffo, E., Belaise, C. (2003)	Psychological aspects of traumatic injury in children and adolescents.	No enfocado en el objetivo
27	Laor, N., Wolmer, L., Spirman, S., Wiener, Z. E. (2003)	Facing war, terrorism, and disaster: toward a child-oriented comprehensive emergency care system.	No es una revisión
28	Miller, L. (2003)	Family therapy of terroristic trauma: Psychological syndromes and treatment strategies	No enfocado en la población
29	Pearn, J. (2003)	Children and war	No enfocado en el objetivo
30	Ermann, M (2004)	Children of World War II - 60 Year after	No enfocado en el objetivo
31	Myers-Walls, J. A. (2004)	Children as victims of war and terrorism	No enfocado en el objetivo
32	Nagao, K. (2004)	War and trauma in children: (II) trauma and its characteristics	No es una revisión.
33	Nagao, K. (2004)	War and trauma in children: (I) war and its victims	No es una revisión.
34	Caffo, E., Forresi, B., Lievers, L. S. (2005)	Impact, psychological sequelae and management of trauma affecting children and adolescents	No enfocado en el objetivo
35	Fazel, M., Wheeler, J., Danesh, J. (2005)	Prevalence of serious mental disorder in 7000 refugees resettled in western countries: A systematic review	No enfocado en el objetivo (revisión de encuestas)
36	Vickers, B. (2005)	Cognitive model of the maintenance and treatment of post-traumatic stress disorder applied to children and adolescents	No es una revisión.
37	Bader, K., Schäfer, V. (2007)	Sleep disturbances following traumatic experiences in childhood and adolescence: A review	No enfocado en el objetivo
38	Kanji, Z., Drummond, J., Cameron, B. (2007)	Resilience in Afghan children and their families: a review	Artículo no encontrado y no hay respuesta de autores.
39	Kanji, Z., Drummond, J., Cameron, B. (2007)	Clinical characteristics and efficacious treatment of post-traumatic stress disorder in children and adolescents	No es una revisión.
40	Lüscher, K., Heuft, G. (2007)	Ambivalence - strain - trauma	No enfocado en la población
41	Bonanno, G. A., Mancini, A. D. (2008)	The human capacity to thrive in the face of potential trauma	No enfocado en el objetivo
42	Castellanos-Obregón, J. M., Torres Silva, W. F. (2008)	A review of academic production on political violence in Colombia to trace the place of youths	No enfocado en el objetivo
43	Cummings, E. M., Goeke-Morey, M. C., Schermerhorn, A. C., Merrilees, C. E., Cairns, E. (2009)	Children and political violence from a social ecological perspective: implications from research on children and families in Northern Ireland	No enfocado en el objetivo
44	Ermann, M., Pflichthofer, D., Kamm, H. (2009, December)	Children of Nazi Germany 60 years on	No enfocado en el objetivo
45	Quiroga, J. (2009)	Torture in children	No enfocado en el objetivo
46	Tufnell, G., DeJong, M. (2009)	Stress and post-traumatic stress disorder	No enfocado en el objetivo
47	Benjet, C. (2010)	Childhood adversities of populations living in low-income countries: prevalence, characteristics, and mental health consequences	No enfocado en el objetivo
48	Robjant, K., Fazel, M. (2010)	The emerging evidence for Narrative Exposure Therapy: a review	No enfocado en el objetivo
49	Mundt, A., Wünsche, P., Heinz, A., Pross, C. (2011)	Trauma therapy in crisis and disaster areas--a critical review of standardized interventions such as narrative exposure therapy	No es una revisión.
50	M Rothe, E., J Pumariega, A., Sabagh, D. (2011)	Identity and acculturation in immigrant and second generation adolescents	No enfocado en el objetivo
51	De Burgh HT, White CJ, Fear NT, Iversen AC. The impact of deployment to Iraq or Afghanistan on partners and wives of military personnel. (2011).	The impact of deployment to Iraq or Afghanistan on military children: a review of the literature	No enfocado en el objetivo
52	Carlson, B. E., Cacciatore, J., Klimek, B. (2012)	A risk and resilience perspective on unaccompanied refugee minors	No es una revisión.
53	Ermann, M. (2012)	Germans reporting about their childhood in the WWII and the Nazi era	No es una revisión.
54	Coren, E., Hossain, R., Pardo, J. P., Veras, M. M., Chakraborty, K., Harris, H., Martin, A. J. (2013)	Interventions for promoting reintegration and reducing harmful behaviour and lifestyles in streetconnected children and young people	No enfocado en el objetivo
55	Danieli, Y. (2013)	Sharing knowledge and shared care	No enfocado en el objetivo
56	Gillies, D., Taylor, F., Gray, C., O'Brien, L., D'Abrew, N. (2013)	Psychological therapies for the treatment of post-traumatic stress disorder in children and adolescents	No enfocado en el objetivo
57	Gillies, D., Taylor, F., Gray, C., O'Brien, L., D'Abrew, N. (2013)	The use of child soldiers in war with special reference to Sri Lanka	No enfocado en el objetivo
58	Lieberman, A. F., Van Horn, P. (2013)	Infants and young children in military families: a conceptual model for intervention	No enfocado en el objetivo
59	Musisi, S., Akena, D., Nakimuli-Mpungu, E., Abbo, C., Okello, J. (2013)	Neuropsychiatric perspectives on nodding syndrome in northern Uganda: a case series study and a review of the literature	No enfocado en el objetivo
60	Orlowski, H. V., Klauer, T., Freyberger, H. J., Seidler, G. H., Kuwert, P. (2013)	The psychology of being unaccounted for, based on the example of children of missing German soldiers from World War II	Artículo no encontrado y no hay respuesta de autores.
61	Smith, R. C., Chun, R. S., Michael, R. L., Schneider, B. J. (2013)	Operation BRAVE families: A preventive approach to lessening the impact of war on military families through preclinical engagement	No enfocado en el objetivo
62	Wadsworth, S. M. (2013)	Understanding and supporting the resilience of a new generation of combat-exposed military families and their children	No enfocado en el objetivo
63	Erdheim, M. (2014)	Adolescence and migration	No es una revisión.
64	Gewirtz, A. H., Zamir, O. (2014)	The impact of parental deployment to war on children: the crucial role of parenting	No enfocado en el objetivo
65	Lambert, J. E., Holzer, J., Hasbun, A. (2014)	Association between parents' PTSD severity and children's psychological distress: a meta-analysis	No enfocado en el objetivo
66	Klin, A., Wetherby, A. M., Woods, J., Saulnier, C., Stapel-Wax, J., Klaiman, C., Bearss, K. (2015)	Toward innovative, cost-effective, and systemic solutions to improve outcomes and well-being of military families affected by autism spectrum disorder	No enfocado en el objetivo
67	Pietraszewski, D., Shaw, A. (2015)	Not by strength alone: children's conflict expectations follow the logic of the asymmetric war of attrition	No enfocado en el objetivo
68	Pfefferbaum B, Jacobs AK, Nitiéma P, Everly GS (2015)	Child debriefing: a review of the evidence base	No enfocado en el objetivo
69	Gillies, D., Maiocchi, L., Bhandari, A. P., Taylor, F., Gray, C., O'Brien, L. (2016)	Psychological therapies for children and adolescents exposed to trauma	No enfocado en el objetivo
70	McAlpine, A., Hossain, M., Zimmerman, C. (2016)	Sex trafficking and sexual exploitation in settings affected by armed conflicts in Africa, Asia and the Middle East: systematic review	No enfocado en la población
71	Masodkar, K., Johnson, J., Peterson, M. J. (2016)	A review of posttraumatic stress disorder and obesity: Exploring the link	No enfocado en la población
72	Pfefferbaum B, Nitiéma P, Jacobs AK, Noffsinger MA, Wind LH, Allen SF (2016)	Review of coping in children exposed to mass trauma: Measurement tools, coping styles, and clinical implications	No enfocado en el objetivo
73	Slone, M., Shur, L., Gilady, A. (2016)	Youth exposed to terrorism: The moderating role of ideology	No enfocado en el objetivo
74	Tierney, D., Bolton, P., Matanu, B., Garasu, L., Barnabas, E., Silove, D. (2016)	The mental health and psychosocial impact of the Bougainville Crisis: A synthesis of available information	No enfocado en la población
75	Wainryb, C., Bourne, S. (2016)	And I Shot Her: On War, and the Creation of Inequities in the Development of Youths' Moral Capacities	No es una revisión.
76	Ba, I., Bhopal, R. S. (2017)	Physical, mental and social consequences in civilians who have experienced war-related sexual violence: a systematic review (1981-2014)	No enfocado en la población
77	Frost, A., Boyle, P., Autier, P., King, C., Zwijnenburg, W., Hewitson, D., Sullivan, R. (2017)	The effect of explosive remnants of war on global public health: a systematic mixed-studies review using narrative synthesis	No enfocado en la población
78	Meiser-Stedman, R., Allen, L. R. (2017)	Start as you mean to carry on: the emerging evidence base for the treatment of conflict-related mental health difficulties in children and adolescents	No es una revisión.
79	ISSOP Migration Working Group. (2018)	ISSOP position statement on migrant child health	No enfocado en el objetivo
80	Purgato, M., Gastaldon, C., Papola, D., Van Ommeren, M., Barbui, C., Tol, W. A. (2018)	Psychological therapies for the treatment of mental disorders in lowand middle-income countries affected by humanitarian crises	No enfocado en el objetivo
81	Sepahvand, H., Hashtjini, M. M., Salesi, M., Sahraei, H., Jahromi, G. P. (2019)	Prevalence of post-traumatic stress disorder (PTSD) in Iranian population following disasters and wars: A systematic review and meta-analysis	No enfocado en la población
82	Tabares, G., Stiths, A. (2019)	Prosociality. Current status of research in colombia	No enfocado en el objetivo
